# A thioredoxin-dependent peroxiredoxin Q from *Corynebacterium glutamicum* plays an important role in defense against oxidative stress

**DOI:** 10.1371/journal.pone.0192674

**Published:** 2018-02-13

**Authors:** Tao Su, Meiru Si, Yunfeng Zhao, Yan Liu, Shumin Yao, Chengchuan Che, Can Chen

**Affiliations:** 1 College of Life Sciences, Qufu Normal University, Qufu, Shandong, China; 2 School of Geography And Tourism, Qufu Normal University, Rizhao, Shandong, China; 3 College of Life Science and Agronomy, Zhoukou Normal University, Zhoukou, Henan, China; Kyoto Daigaku, JAPAN

## Abstract

Peroxiredoxin Q (PrxQ) that belonged to the cysteine-based peroxidases has long been identified in numerous bacteria, but the information on the physiological and biochemical functions of PrxQ remain largely lacking in *Corynebacterium glutamicum*. To better systematically understand PrxQ, we reported that PrxQ from model and important industrial organism *C*. *glutamicum*, encoded by the gene *ncgl*2403 annotated as a putative PrxQ, played important roles in adverse stress resistance. The lack of *C*. *glutamicum prxQ* gene resulted in enhanced cell sensitivity, increased ROS accumulation, and elevated protein carbonylation levels under adverse stress conditions. Accordingly, PrxQ-mediated resistance to adverse stresses mainly relied on the degradation of ROS. The physiological roles of PrxQ in resistance to adverse stresses were corroborated by its induced expression under adverse stresses, regulated directly by the stress-responsive ECF-sigma factor SigH. Through catalytical kinetic activity, heterodimer formation, and bacterial two-hybrid analysis, we proved that *C*. *glutamicum* PrxQ catalytically eliminated peroxides by exclusively receiving electrons from thioredoxin (Trx)/thioredoxin reductase (TrxR) system and had a broad range of oxidizing substrates, but a better efficiency for peroxynitrite and cumene hydroperoxide (CHP). Site-directed mutagenesis confirmed that the conserved Cys49 and Cys54 are the peroxide oxidation site and the resolving Cys residue, respectively. It was also discovered that *C*. *glutamicum* PrxQ mainly existed in monomer whether under its native state or functional state. Based on these results, a catalytic model of PrxQ is being proposed. Moreover, our result that *C*. *glutamicum* PrxQ can prevent the damaging effects of adverse stresses by acting as thioredoxin-dependent monomeric peroxidase could be further applied to improve the survival ability and robustness of the important bacterium during fermentation process.

## Introduction

Reactive oxygen species (ROS) and reactive nitrogen species (RNS) are highly active compounds that are unavoidably generated during normal cell metabolism and also overdone under adverse conditions. ROS includes hydrogen peroxide (H_2_O_2_), superoxide (O_2_^.^−), and the hydroxyl radical (OH·), and RNS includes NO and its derivate peroxynitrite (ONOO). Excessive highly active compounds gives rise to oxidative damage to macromolecules such as DNA, protein, nucleic acid, as well as lipid, eventually causing loss of membrane integrity and cell death [1]. Consequently, several routes of defense mechanisms have been developed, including those based on nonenzymatic elements such as glutathione (GSH) and vitamin C and antioxidant enzymes such as catalase (CAT), superoxide dismutase (SOD), and peroxidase [2–4].

Peroxiredoxins (Prxs) have received considerable attention in recent years as a growing family of thiol-specific non-heme antioxidant peroxidases detoxifing hydrogen peroxide, alkyl peroxides, and peroxinitrite [5]. Based on abundant sequence homology and structural similarity analyses, they have been divided into six subfamilies: AhpC/Prx1, Prx6, Prx5, Tpx, PrxQ-BCP, and AhpE [5–8]. Alternatively, they can be classified into three types according to their distinct catalytic mechanisms: typical 2-Cys (Prx1 and AhpC), atypical 2-Cys (Tpx, PrxQ, and Prx5), and 1-Cys Prxs (Prx6 and AhpE) [9–11]. All types of Prx share a common catalytic step involving a strictly conserved peroxidatic cysteine (C_P_) in a “fully folded” (FF) conformation, which attacks peroxide substrate to be oxidized into the Cys-sulfenic acid (C_P_-SOH) [9–10]. To regenerate the active form of Prx and go on another catalytic cycle, the Cys-sulfenic acid (C_P_-SOH) must be reduced to a thiol and recycled to the C_P_. For 2-Cys Prxs, the Cys sulfenic acid (Cys-SOH) intermediate reacts with the resolving Cys (C_R_) of the same subunit (atypical 2-Cys subfamily) or another subunit (typical 2-Cys subfamily) to form an intramolecular disulfide bond-containing monomer or intermolecular disulfide bond-containing dimmer [9–10]. The disulfide bond is then reduced by an external reducing substrate, usually thioredoxins (Trx). Unlike 2-Cys Prxs, the Cys-SOH derivative of 1-Cys Prxs is regenerated by thiol-containing electron donors such as Trx, GSH, glutaredoxin (Grx) or glutathione transferase π [12–14]. Among Prxs, PrxQ subfamily is speculated to be the most ancestral-like protein yet is among the most complex characteristic and the least systematically characterized. PrxQs usually exists only in monomeric state with an intramolecular disulfide bond between two Cys residues separated by only four amino acids and were reduced by Trx [15]. However, recently Limauro *et al*. found that *Sulfolobus solfataricus* PrxQ4 (SsPrxQ4) was present by a dimer with an intermolecular bridge [16]. Some reports also showed there was Grx-dependent and C_R_-lacking PrxQs, and mutagenesis studies on C_R_-containing PrxQ indicated that this protein may be functional with only the catalytic Cys (C_P_) [17–18]. Besides, PrxQ appears to be vital for the viability of *Escherichia coli* and *Helicobacter pylori* but not *Arabidopsis *under oxidative stresses [4, 17, 19–21]. More importantly, the physiological function of resistance to environmental stresses, physiological electron donor, substrate specificity of PrxQs are largely neglected. Therefore, currently existing results are contradictory, unilateral, and unsystematic, which makes PrxQ deserve to be further studied.

The Gram-positive bacterium *Corynebacterium glutamicum*, an important industrial microorganism for producing amino acids, nucleotides and other bio-based chemicals, inevitably encounters oxidative stress during fermentation. To deal with such hostile conditions, this species has developed multiple resistance systems, including the major LMW thiol, mycothiol (MSH; 1D-myo-inosityl-2-(N-acetyl-L-cysteinyl) amido-2-deoxy-γ-D-glucopyranoside) and ROS-scavenging enzymes such as CAT, SOD, organic hydroperoxide reductase (Ohr), mycothiol peroxidase (MPx), and methionine sulfoxide reductase A (MsrA), thiol peroxidase (Tpx) [22–27]. Although PrxQs had been investigated in many organisms, much less is known about its physiologic and biochemical role in protecting *C*. *glutamicum* from peroxide toxicity [15–18, 22–30]. Therefore, this study was performed to determine whether *C*. *glutamicum* PrxQ affects the survival of *C*. *glutamicum* under adverse stress. Moreover, biochemical studies to investigate the catalytic mechanisms, the preferential substrates, and the molecular states of *C*. *glutamicum* PrxQ were characterized.

## Materials and methods

### Bacterial strains and culture conditions

The bacterial strains and plasmids used in this study are listed in S1 Table. *C*. *glutamicum* and *Escherichia coli* strains were cultured in Luria-Bertani (LB) broth aerobically on a rotary shaker (220 rpm) or on LB plates at 30°C or 37°C, respectively, as previously reported [25, 31]. To construct the Δ*prxQ* in-frame deletion mutants, the pK18*mobsacB*-Δ*prxQ* plasmids were transferred into *C*. *glutamicum* by electroporation and chromosomal integration was selected by single crossover on LB agar plates containing 25 μg/ml kanamycin and 40 μg/ml nalidixic acid. After the above kanamycin-resistant (Km^R^) colonies were cultured overnight in LB for a second cross-over, the Δ*prxQ* deletion mutants were subsequently screened on LB agar plates containing 20% sucrose and 40 μg/ml nalidixic acid and then confirmed by PCR and DNA sequencing as previously described [25, 31]. For complementation in relevant *C*. *glutamicum* strains, the pXMJ19 derivatives were transferred into relevant *C*. *glutamicum* strains by electroporation and the expression in *C*. *glutamicum* was induced by addition of 0.5 mM isopropyl β-D-1-thiogalactopyranoside (IPTG). Sensitivity assays for peroxides were investigated as described [24–27]. To express and purify His_6_- or GST-tagged proteins, these pET28a and GST derivatives were transferred into *E*. *coli* BL21(DE3) strains. Recombinant proteins were obtained as described [24]. Cleavage of the His_6_ tag was performed by adding 10 units of Enterokinase-Max (Invitrogen, Karlruhe, Germany) and incubation at 4°C overnight to conduct subsequent polymerization analysis via PAGE and gel filtration chromatography. Ni-NTA agarose was used to remove the cleaved tag and uncleaved protein from the tag-free protein. All enzymes were purchased from Sigma-Aldrich (St. Louis, MO). All antibiotics were purchased from Gold Biotechnology (Shanghai, China). All chemicals, oxidants, and heavy metals were purchased from aladdin (Shanghai, China). When needed, antibiotics were used at the following concentrations: kanamycin, 50 μg ml^-1^ for *E*. *coli* and 25 μg ml^-1^ for *C*. *glutamicum*; nalidixic acid, 40 μg ml^-1^ for *C*. *glutamicum*; chloramphenicol, 20 μg ml^-1^ for *E*. *coli* and 10 μg ml^-1^ for *C*. *glutamicum*.

### Plasmid construction

For obtaining expression plasmids, the genes encoding for *C*. *glutamicum* thioredoxin 2 (Trx2, NCgl2874), thioredoxin 3 (Trx3, NCgl0289), and peroxiredoxin Q (PrxQ, NCgl2403) were amplified by PCR. The obtained DNA fragments were digested and cloned into similar digested pET28a, pGEX6p-1, or pKT25M vectors, obtaining pET28a-*trx2*, pET28a-*trx3*, pET28a-*prxQ*, pGEX6p-1-*prxQ*, and pKT25M-*prxQ*, respectively.

The plasmid pK18*mobsacB*-Δ*prxQ* was made by overlap PCR with primers listed in S2 Table [32]. Briefly, the 904-bp upstream fragment and the 890-bp downstream fragment of *prxQ* were amplified with primer pairs DPrxQ-F1/DPrxQ-R1 and DPrxQ-F2/DPrxQ-R2, respectively. The upstream and downstream PCR fragments were fused together with the primer pair DPrxQ-F1/ DPrxQ-R2 by overlap PCR [32]. The resulting products were digested with BamH I and Xho I, and inserted into similar digested plasmid pK18*mobsacB* to produce pK18*mobsacB*-Δ*prxQ*.

To produce pXMJ19-*prxQ*, PrxQ-F/PrxQ-R as primers and *C*. *glutamicum* genomic DNA as templates were used. The obtained DNA fragments were digested and cloned into similar digested pXMJ19 vector.

To make the cysteine residue at position 49 of PrxQ into a serine residue (PrxQ:C49S), site-directed mutagenesis was performed by overlap PCR [24]. In brief, the mutant *prxQ*:*C49S* DNA segment was amplified by two rounds of PCR. In the first round of PCR, primer pairs DPrxQ-F1/PrxQ-C49S-R and PrxQ-C49S-F/DPrxQ-R2 were used to amplify segments 1 and 2, respectively. The second round of PCR was performed by using PrxQ-F/PrxQ*-*R as primers and fragment 1 and fragment 2 as templates to get the *prxQ*:*C49S* fragment. The *prxQ*:*C49S* DNA fragment was digested and cloned into similar digested pET28a or pXMJ19 plasmid, obtaining plasmid pET28a-*prxQ*:*C49S* or pXMJ19-*prxQ*:*C49S*. The *prxQ*:*C54S*, *trx1*:*C32S*, *trx1*:*C35S*, *trx2*:*C30S*, *trx2*:*C33S*, *prxQ*:*C49SC54S* fragments were gained using similar procedure as described above and also cloned into plasmid pET28a, pXMJ19, or pUT18CM, obtaining corresponding plasmids. All primers used in this study are listed in S2 Table.

For getting the *lacZ* fusion reporter vector pK18*mobsacB-P*_*prxQ*_::*lacZ*, the fusion of *prxQ* promoter to the *lacZY* reporter gene by overlap PCR was made [32–33]. Firstly, the primers PPrxQ-F1/PPrxQ-R1 and lacZY-F/lacZY-R were used in the first round of PCR to amplify the 700-bp *prxQ* promoter DNA fragments and the *lacZY* DNA fragments, respectively. Secondly, PPrxQ-F1/lacZY-R as primers and the first round PCR products as template were used to perform the second round of PCR, and the resulting fragments were digested with Sal I/Pst I and inserted into similar digested pK18*mobsacB* to obtain the pK18mobsacB*-P*_*prxQ*_::*lacZ* fusion construct [26].

The fidelity of all constructs was confirmed by DNA sequencing (Sangon Biotech, Shanghai, China).

### Catalytic kinetic activity measurement

Peroxidase activity of PrxQ toward hydrogen peroxide (H_2_O_2_), Cumene-OOH (CHP), and *t*-Butyl-OOH (*t*-BOOH) was measured using an NADPH-coupled spectrophotometric method as described [34]. The assays were performed in the reaction mixture (500 μl) containing 50 mM Tris-HCl buffer (pH 8.0), 2 mM EDTA, 250 μM NADPH, 1 μM PrxQ (WT or its variants), and the reduced Trx-generating system (15 μM thioredoxin reductase (TrxR) and 40 μM Trx), or the Mrx1-generating system [500 μM MSH, 15 μM mycothione reductase (Mtr), and 40 μM mycoredoxin 1 (Mrx1)]. After a 5 min preincubation, the addition of 1 mM H_2_O_2_, CHP, or *t*-BOOH started the reaction. The catalytic kinetic parameters for one substrate were acquired by changing its concentration in the case of saturating concentrations of the other substrate (between 0 and 120 μM for Trx or between 0 and 2 mM for peroxide). The decrease of NADPH absorbance was monitored at 340 nm. The activity was determined after subtracting the spontaneous reduction rate detected without PrxQ, and the micromoles amount of NADPH oxidized per second per micromole of enzyme (i.e. turnover number, s^−1^) was calculated by the molar absorption coefficient of NADPH at 340 nm (*ε*_340_) of 6220 M^−1^ cm^−1^. Three independent experiments were performed at each substrate concentration. The *k*_cat_ and *K*_m_ values of PrxQ were acquired from a non-linear fit with the Michaelis-Menten equation by the program GraphPad Prism 5.

### Determination of the reaction activity of PrxQ with peroxynitrite

The reaction rate of PrxQ with peroxynitrite was investigated using a competition approach by measuring the inhibitor effect of increasing PrxQ concentrations on peroxynitrite-mediated HRP oxidation to compound I as described previously [35–39].

### Measurement of intracellular ROS levels and determination of cellular protein carbonylation

Intracellular ROS level was detected using the fluorogenic probe 2′, 7′-dichlorodihydrofluorescein diacetate (DCFH-DA) (Sigma-Aldrich, St. Louis, MI, USA) as described [40]. Protein carbonylation assays were investigated as described previously [25–27, 41].

### Determination of the oligomerization and redox states

The oligomerization state of PrxQ was analyzed with 15% native-PAGE according to the method described previously [26]. The mixtures of the tag-free native PrxQ and the loading buffer containing 250 mM Tris-HCl (pH6.8), 0.5% (W V^-1^) bromophenol blue (BPB) and 50% (V V^-1^) glycerol were separated on 15% PAGE and subsequently stained with Coomassie Brilliant Blue.

The oligomerization state of PrxQ was also analyzed with gel filtration method on Superdex 75 10/300 GL column connected to an FPLC system (GE Healthcare, Piscataway, NJ) as previously described [26]. The tag-free proteins were loaded onto the column pre-equilibrated with 0.15 M NaCl–containing potassium phosphate buffer (50 mM, pH 7.2). The molecular mass of standard proteins used was: aprotinin (6.5 KDa), ribonuclease A (13.7 KDa), carbonic anhydrase (29 KDa), ovalbumin (44 KDa) and conalbumin (75 KDa) (GE Healthcare, Piscataway, NJ). The *K*_av_ values for each of the standard protein were calculated by the equation: *K*_av_ = (*V*_e_–*V*_0_)/(*V*_c_–*V*_0_), where *V*_0_ = column void volume, *V*_e_ = elution volume and *V*_c_ = geometric column volume. The flow rate was stetted as 0.5 ml min^-1^ and the absorbance was monitored at 280 nm. The calibration curve was obtained by the plots of *K*_av_ values from the above standard proteins against the log of the molecular weight of the standard protein. These data were suited with a linear equation. The molecular weight of the unknown protein could be calculated by its measured elution volume, its *K*_av_ value and the calibration curve.

The redox states of PrxQs wild type (WT) or its variants were analyzed as described [26]. Briefly, 10 μM PrxQs incubated with 50 mM DTT (dithiothreitol) or 1 mM H_2_O_2_ for 30 min were mixed with the loading buffer containing DTT and β-mercaptoethanol (for reducing conditions) or 50 mM iodoacetamide (IAM) (for non-reducing conditions), and then separated on reducing- or non-reducing 15% SDS-PAGE.

### Analysis of sulfenic acid and disulfide bond formation

The formation of sulfenic acid was measured by the assays of DTT-treated or H_2_O_2_-treated proteins labeled with 4-chloro-7-nitrobenzofurazan (NBD-Cl) [42]. The formation of disulfide bond was examined with the thiol-reactive probe 4-acetamido-4’-maleimidyldystilbene-2, 2’-disulfonic acid (AMS; Molecular Probes, Eugene, OR) [43].

### Quantitative analysis of sulfhydryl groups

Free sulfhydryl groups in PrxQ WT and its variants were examined with 5, 5’-dithio-bis (2-nitrobenzoic acid) (DTNB) as described [44].

### Examination of heterodimers

The heterodimers assays were determined as described with minor modifications [45]. 20 μM PrxQ variants and 15 μM Trx (WT and its variants) were mixed in TE buffer containing 30 mM Tris-HCl (pH 8.0) and 1 mM EDTA. After the reaction mixture was incubated at room temperature for 15 min, 50 μM H_2_O_2_ was added and then continued to culture at room temperature for 30 min. The resulting samples were resolved on 15% non-reducing SDS-PAGE.

### Construction of chromosomal fusion reporter strains and β-galactosidase activity analysis

Chromosomal fusion reporter strains were obtained as described [33]. Briefly, the *lac*Z fusion reporter plasmid pK18*mobsacB-P*_*prxQ*_::*lac*Z was transferred into the relavant *C*. *glutamicum* by electroporation, and the pK18*mobsacB-P*_*prxQ*_::*lac*Z fusion reporter strain was selected on LB agar plates containing kanamycin. β-galactosidase activity was determined with *o*-nitrophenyl-β-galactoside (ONPG) as substrate [46].

### Electrophoretic mobility shift assay (EMSA)

EMSA was performed according to the method described previously [47]. In brief, *P*_*prxQ*_ fragments were obtained by *prxQ* promoter region of pK18*mobsacB*-*P*_*prxQ*_::*lac*Z reporter vector as templates and PPrxQ-F2/PPrxQ-R as primers. Each 10-μl EMSA reaction solutions contained 20 mM Tris-HCl (pH 7.4), 4 mM MgCl_2_, 100 mM NaCl, 1 mM DTT, 10% (W V^-1^) glycerol, 20 ng PPrxQ DNA fragments, and different concentrations of His_6_-SigH (0–4 μg). After reaction for 20 min, the protein-probes mixture was separated on a 6% native polyacrylamide gel and then stained with SYBR Green (Promega, Fitchburg, WI). Fragments from the *prxQ* coding region obtained with primer pair Control-F and Control-R instead of the *prxQ* promoter were added to the binding assays to act as negative controls.

### Western blot analysis

Western blot analysis was performed as described previously [32]. Samples subjected on SDS-PAGE were transferred onto polyvinylidene fluoride (PVDF) membranes. After blocking with 4% (w v^-1^) milk for 2 h at room temperature, membranes were incubated with the primary antibody at 4°C overnight: anti-His; anti-DNP; anti-GST (Zhongshan Gold Bridge, Beijing). The blots were washed with 0.2% (v v^-1^) Tween 20-containing PBST buffer and then incubated with horseradish peroxidase conjugated secondary antibody (Shanghai Genomics Inc., Shanghai, China). The protein bands were visualized with ECL plus kit (GE Healthcare, Piscataway, NJ).

### Quantitative RT-PCR analysis

Quantitative RT-PCR analysis (7500 Fast Real-Time PCR; Applied Biosystems, Foster City, CA) was performed as described previously [48]. The primers used are listed in S2 Table. To obtain standardization of results, the relative abundance of 16S rRNA was used as the internal standard.

### GST pull-down assay

The GST pull-down assay was performed as described previously [49]. Retained proteins were examined after SDS-PAGE using the anti-His antibody (Millipore, MA, USA).

### Statistical analysis

Statistical analyses of survival rate, ROS level, catalytic kinetic activity and transcription level were determined with paired two-tailed Student’s t-test. GraphPad Prism Software was used to carry out statistical analyses (GraphPad Software, San Diego California USA).

## Results

### Indentification of *prxQ* gene from *C*. *glutamicum* genome and oligomeric state analysis

**Fig 1:**
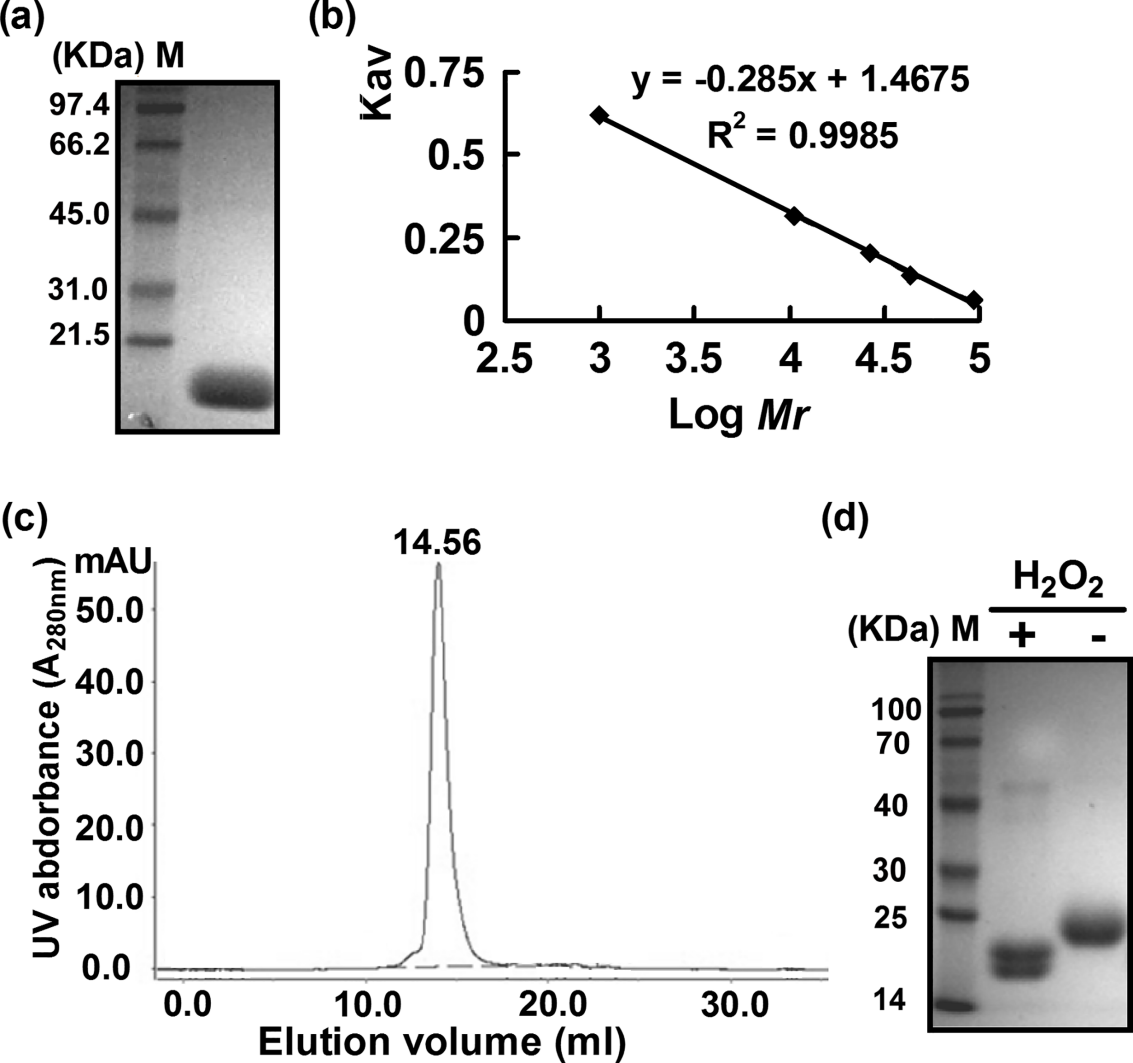
Determination of the molecular weights of native and oxidized peroxiredoxin Q (PrxQ). (**a**) The purified recombinant PrxQ was mixed with loading buffer containing 250 mM Tris-HCl (pH 6.8), 0.5% (m V^-1^) bromophenol blue, and 50% (V V^-1^) glycerol, resolved on 15% polyacrylamide gel electrophoresis (PAGE) (pH 8.8), and then stained with Coomassie Brilliant Blue. M, molecular weight markers. (**b**) Molecular weight standard curve. (**c**) Gel filtration of the native PrxQ. Molecular weight of the purified PrxQ was estimated using the above molecular weight standard curve. (**d**) Redox response of PrxQ. 50 mM dithiothreitol (DTT)-treated proteins (10 μM) were incubated with (+) or without (−) 1 mM H_2_O_2_, and then the resulting samples were resolved on 15% non-reducing SDS-PAGE.

A blast search of *C*. *glutamicum* genome with a consensus PrxQ sequence identified a gene annotated as *prxQ* (*ncgl2403*) encoding a protein of 158 amino acids with a theoretical molecular mass of 17 kDa. The *C*. *glutamicum* PrxQ showed 49%, 39% and 34% amino acid identities with *Mycobacterium tuberculosis* PrxQ B, *Synechococcus* PrxQ, and *Escherichia coli* PrxQ, respectively (S1 Fig). Previous studies showed that PrxQ usually behave as atypical 2-Cys Prx, which means that it mainly exists in monomer whether under its native state or functional state [15]. To determine the oligomeric form exhibited by *C*. *glutamicum* PrxQ, it was expressed in *E*. *coli* BL21 (DE3), purified by homogeneity, and cleaved with Enterokinase-Max to remove His_6_ tag. As shown in Fig 1A, the tag-free recombinant native PrxQ protein showed a single band with a molecular size of approximately 17.0 KDa on 15% polyacrylamide gel electrophoresis (PAGE) (pH 8.8). To further confirm the oligomeric properties of the tag-free native PrxQ, we performed analytical gel filtration chromatography, which enables the molecular mass to be determined independently of the molecular shape. In the gel filtration chromatogram, a sharp peak occurred at an elution volume of 14.56 ml (Fig 1C). Using the standard curve (Fig 1B), the molecular mass of PrxQ was estimated to be 17 KDa, corresponding well to the molecular mass of PrxQ deduced from its amino acid sequence, indicating that the PrxQ eluted from the column was entirely monomeric. Next, we determined the oxidized form of purified wild-type (WT) PrxQ under H_2_O_2_ treatment. As shown in Fig 1D, the oxidized PrxQ migrated as a monomer as judged by its behavior on 15% non-reducing SDS-PAGE. Notably, the monomeric forms of H_2_O_2_-treated PrxQ showed two bands, one of which migrated more rapidly. This would be agreement with the formation of the intramolecular disulfide bonds imparting the proteins more compact configuration and more rapid migration. Together, these findings showed that *C*. *glutamicum* PrxQ mainly exists in monomer both under reduced and oxidized states, similar to polpar PrxQ and *M*. *Tuberculosis* PrxQ B [29–30].

### Roles of PrxQ in adverse stresses responses in *C*. *glutamicum*

**Fig 2:**
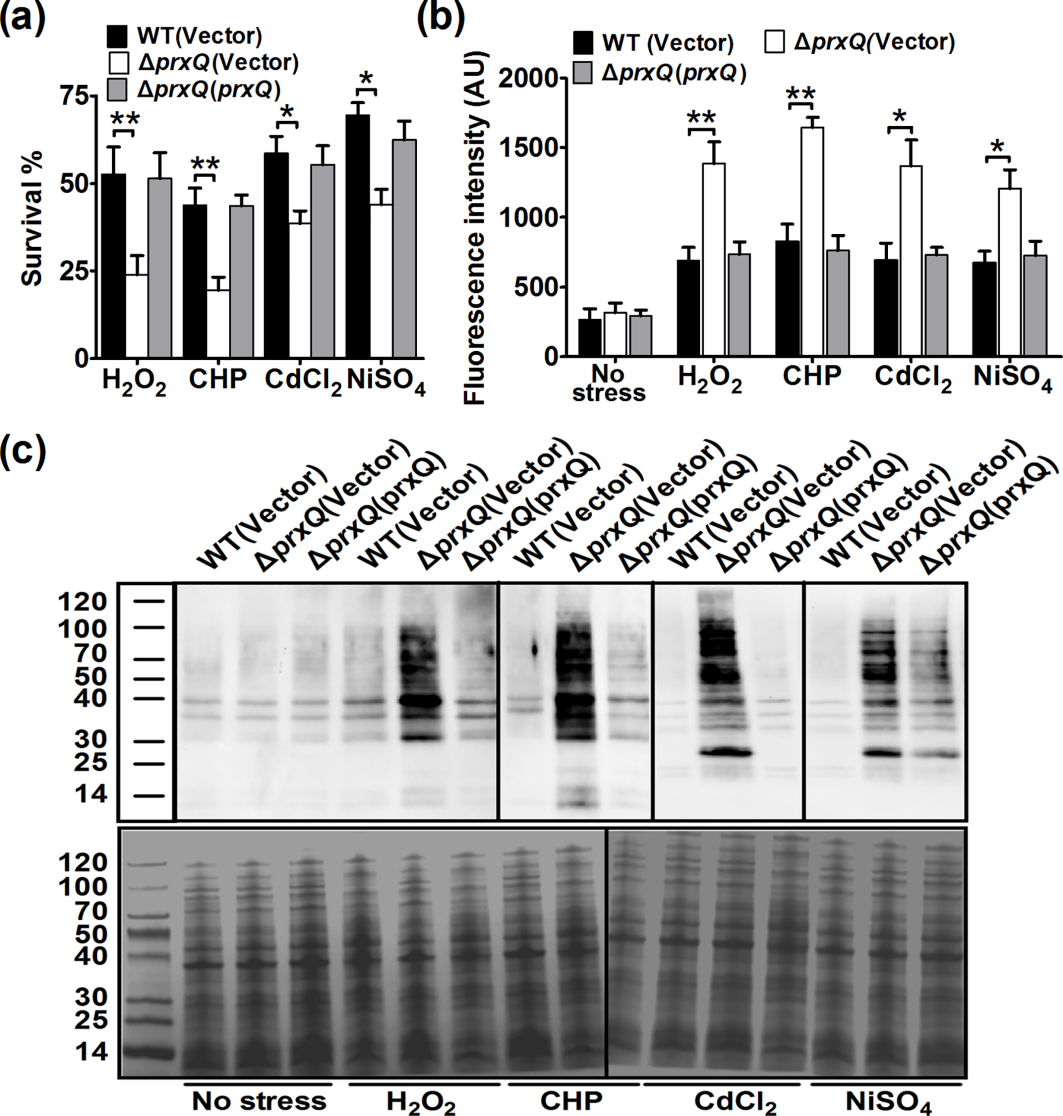
Peroxiredoxin Q (PrxQ) is required for cellular resistance to diverse stresses in *Corynebacterium glutamicum*. *C*. *glutamicum* wild-type WT(Vector), Δ*prxQ*(Vector), and Δ*prxQ*(*prxQ*) strains grown to stationary phase were exposed to the adverse stresses for 30 min. (**a**) The mutant lacking PrxQ was highly sensitive to adverse stresses. The viability of the cells was determined. Mean values with standard deviations (error bars) from at least three repeats are shown. **: *P*≤0.01; *: *P*≤0.05. (**b**) Deletion of *prxQ* led to accumulation of intracellular ROS. The intracellular levels of ROS were determined with the DCFH-DA probe after exposure of stationary phase strains to adverse stresses. **: *P*≤0.01; *: *P*≤0.05. (**c**) The mutant lacking PrxQ had enhanced protein carbonyl levels under adverse stresses. 20 μg of each DNPH-derivatized protein were loaded and electrophoresis was conducted on a 15% SDS-PAGE gel. The protein carbonyl levels were measured with anti-dinitrophenyl antibody (Upper panel). A parallel run was stained with Coomassie Brilliant Blue (Bottom panel). Similar results were obtained in three independent experiments, and the data shown are from one representative experiment.

For evaluating the physiological role of PrxQ in resistance to adverse stresses, we compared the viability of the wild type (WT) *C*. *glutamicum*, Δ*prxQ* mutant, and complementary strains in the presence of H_2_O_2_ (100 mM), cumene hydroperoxide (CHP, 11 mM), CdCl_2_ (0.3 mM), and NiSO_4_ (6 mM). As shown in Fig 2A, deletion of *prxQ* gene obviously decreased the resistance of *C*. *glutamicum* to these conditions compared with the wild type. However, the hypersensitive phenotype of the Δ*prxQ* mutant to adverse stresses was almost completely reversed by complementation with the wild type *prxQ* gene, which was very similar to the case for the WT(Vector) strain. Hence, PrxQ is critical for protection against adverse stresses.

It has been proposed that various stresses, such as heavy metals, antibiotics, alkylating agents, and acids, can cause cellular death by a prevalent oxidative damage mechanism that depends on the ROS levels [1]. Previous studies showed that peroxiredoxin cleared ROS [5, 50], so we examined whether PrxQ played a role in removing ROS under ROS-inducing stress conditions (Fig 2B). For all these stresses tested, WT(Vector) strain showed obviously lower ROS levels as compared to that in the Δ*prxQ*(Vector) strain. However, the complementation strain showed a greater decrease in ROS contents than the Δ*prxQ*(Vector) strain, and showed the same level of ROS generation as the WT(Vector) strain. ROS escaping from the antioxidant defense system are more easily to react with Cys residue of proteins, leading to the reversible formation of molecular disulfide bonds and mixed disulfide bond, or the irreversible formation of sulfoxidation products and protein carbonylation [51–52]. To test whether PrxQ protects cells through reducing the oxidative damage suffered by proteins under oxidative stress, carbonyl groups on total proteins isolated from WT(Vector), Δ*prxQ*(Vector), and Δ*prxQ*(*prxQ*) strains under exposure to adverse stresses were derivatized with 2, 4-dinitrophenyl hydrazine (DNPH) and detected by Western blot using the anti-DNP antibody. As shown in Fig 2C, the Δ*prxQ*(Vector) strain showed more increase in the carbonylation level of protein extracts as compared to that in the WT(Vector) strain. However, it is noteworthy that the carbonylation level in the complementary strain was almost completely reduced up to the level of WT(Vector) strain.

### Peroxidase activities of PrxQ using Trx as an electron donor

**Fig 3:**
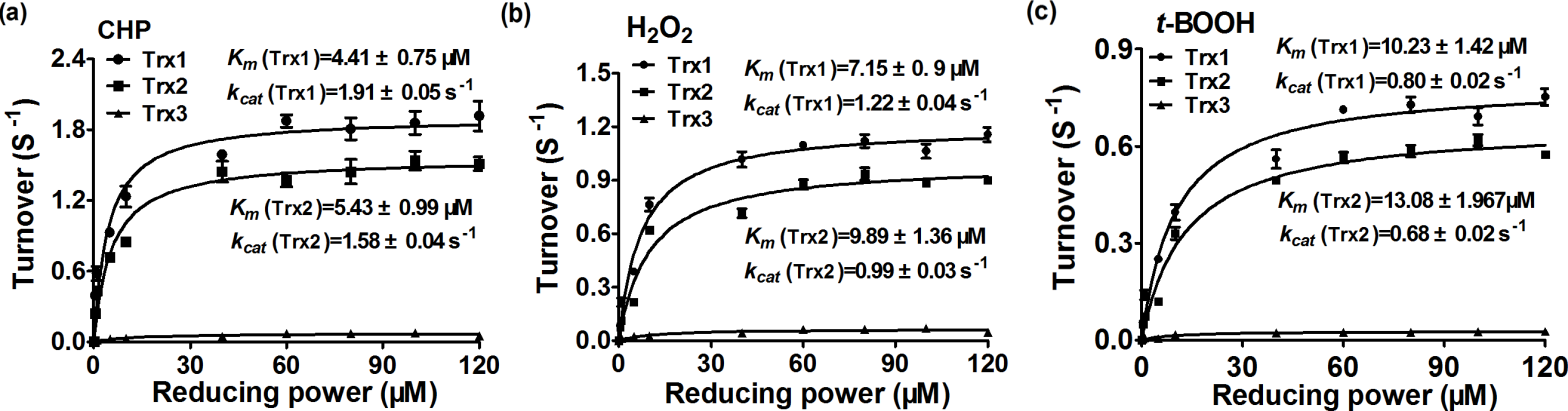
Reductase activities of peroxiredoxin Q (PrxQ) using thioredoxin using Trx as an electron donor. (**a-c**) The Michaelis-Menten plots of PrxQ activity versus different thioredoxins by NADPH-coupled spectrophotometric method. The reaction mixtures containing 50 mM Tris-HCl buffer (pH 7.5), 2 mM EDTA, 250 μM NADPH, 1 μM PrxQ, 15 μM TrxR, 1 mM peroxides, and 0–120 μM Trx1(a), or 1 0–120 μM Trx2 (b), or 0–120 μM Trx3 (c), or 0–2 mM peroxides and 40μM Trx1 (d), or 0–2 mM peroxides and 40μM Trx2 (e). The data were analyzed by nonlinear regression using the program GraphPad Prism 5 and are presented as means of the values obtained from three independent assays.

Although the putative *C*. *glutamicum* PrxQ was suspected to act as an atypical 2-Cys Prx that receives electrons from Trx/TrxR system to reduce peroxides, the experimental evidence was lacking. To explore this activity, we performed an *in vitro* assay system by adding different oxidants as substrate and the coupled NADPH oxidation by Trx/TrxR system was monitored at 340 nm. Since *C*. *glutamicum* contains three alternative Trxs, i.e. Trx1, Trx2 and Trx3, we examined the ability of PrxQ to use each reducing equivalents to sustain peroxidase activity at saturating concentration of substrates and different concentration reducing power (0–120μM). As shown in Fig 3, the apparent affinity of PrxQ towards Trx1 was obtained in steady-state conditions, mildly higher than the value determined with Trx2. For example, the *k*_cat_ values of PrxQ for CHP with Trx1 and Trx2 systems were 1.91 ± 0.05 s^-1^ and 1.58 ± 0.04 s^-1^, respectively, while the respective *K*_m_ values were 4.41 ± 0.75 μM and 5.43 ± 0.99 μM. These correspond to a catalytic efficiency of 4.3×10^5^ M^-1^ · s^-1^ and 2.9×10^5^ M^-1^ · s^-1^, respectively. Unlike Trx1 and Trx2, PrxQ cannot use Trx3 as an electron donor. So Trx1 and Trx2 were active with an apparent efficiency order Trx1 > Trx2. Moreover, experiments carried out with different Trx concentrations indicated that the reaction saturate at Trx concentrations between 4 and 14 μM.

We further asked whether PrxQ was able to reduce peroxides via the mycoredoxin 1/ MSH/mycothione reductase (Mxr1/MSH/Mtr) pathway, a novel electron transfer pathway identified in MSH producing high-(G+C)-content Gram-positive *Actinobacteria* that functional equivalent to the widely distributed glutaredoxin/glutathione/ glutathione reductase (Grx/GSH/GR) pathway [23]. However, no NADPH consumption was observed by using the Mrx1/Mtr/MSH reducing system (S2 Fig), indicating that this system cannot provide electrons to PrxQ. Together, these results showed that although PrxQ can employ the two Trxs regeneration systems in reducing oxidant, it has higher affinity for Trx1. That is, Trx1 was the most efficient electron donor for PrxQ.

**Fig 4:**
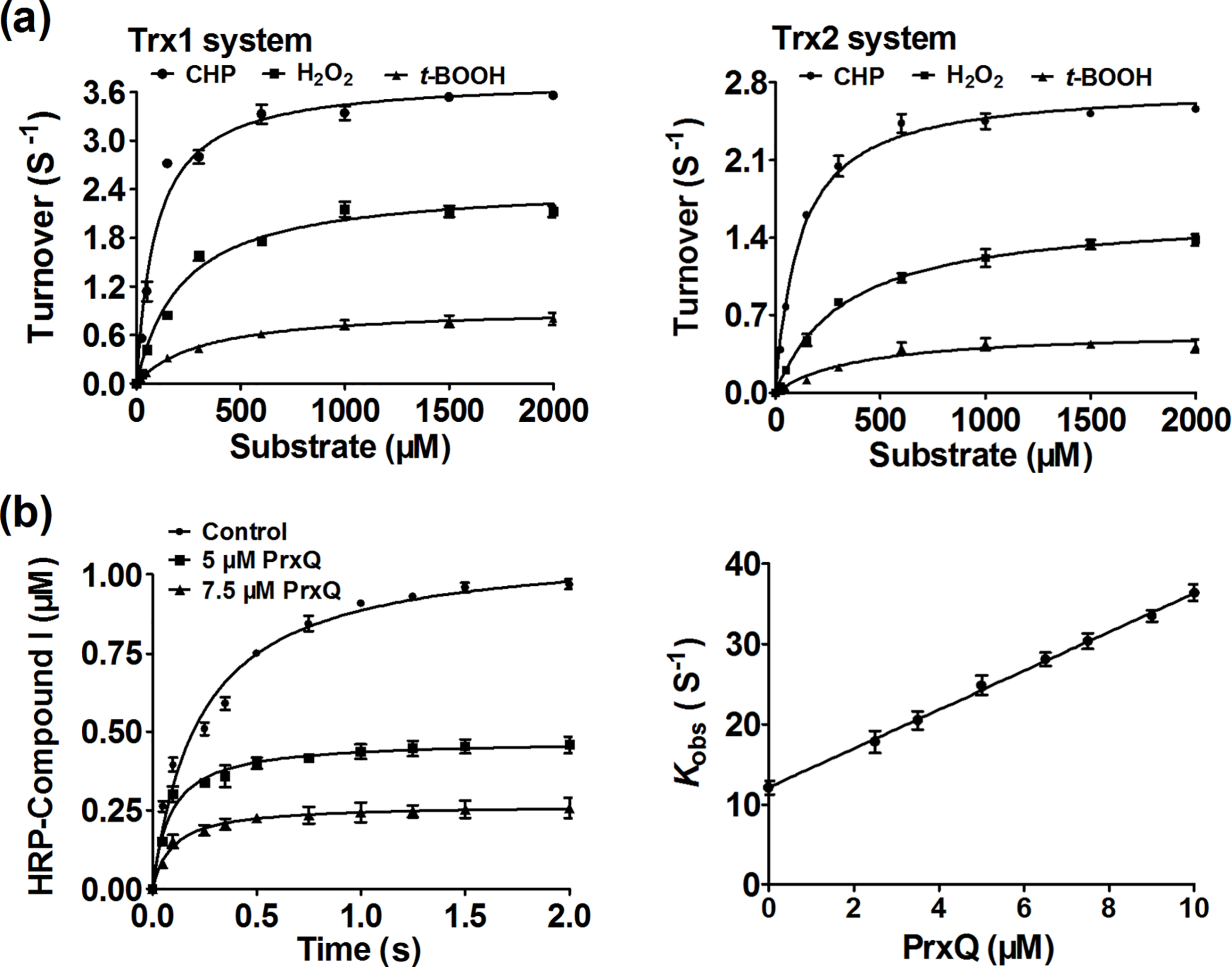
Oxidizing substrate of PrxQ. (**a**) The Michaelis-Menten plots of PrxQ activity versus different substrates by NADPH-coupled spectrophotometric method.The reaction mixtures containing 50 mM Tris-HCl buffer (pH 7.5), 2 mM EDTA, 250 μM NADPH, 1 μM PrxQ, 15 μM TrxR, and 40 μM Trx1 and 0–2 mM peroxides (upper left), or 40 μM Trx2 and 0–2 mM peroxides (upper right). The data were analyzed by nonlinear regression using the program GraphPad Prism 5 and were presented as means of the values obtained from three independent assays. (**b**) Kinetics of peroxynitrite reduction by PrxQ. Peroxynitrite (1 μM) in 10 mM NaOH was rapidly mixed with HRP (5 μM) in the absence or presence of increasing concentrations of PrxQ in 100 mM sodium phosphate buffer (pH 7.4) at 25°C. The inset shows the experimental traces corresponding to HRP compound I formation without PrxQ (control) and with different concentration of PrxQ (0.0, 5.0, 7.5 μM) (lower left). Experimental data were fitted to single exponentials from which observed rate constants of HRP compound I formation were determined. The latter were plotted against PrxQ concentrations (lower right). The data were presented as means of the values obtained from three independent assays.

Next, enzymatic activities of PrxQ toward various oxidizing substrates were analyzed using the Trx/TrxR system as the reducing power (Fig 4 and S3 Table). Results showed that the catalytic efficiency (*k*_cat_/*K*_peroxide_) of PrxQ towards H_2_O_2_, CHP, and *t*-BOOH, is quite variable with values of 1.2×10^3^ M^−1^ · s^−1^ ~ 4×10^4^ M^−1^ · s^−1^ (Fig 4A and 4B). The distinct difference observed with CHP was relevant with a difference in the apparent substrate affinity, with *K*_m_ values of about 93.74~115.8 μM for CHP, and 220.9~333.4 and 316.8~473.9 μM for H_2_O_2_ and *t*-BOOH. The above values obtained from *C*. *glutamicum* PrxQ showed comparability with those from Poplar PrxQ and *E*. *coli* BCP, indicating that these enzymes have very similar properties [29,17].

Apart from H_2_O_2_, CHP, and *t*-BOOH, peroxynitrite has also been reported for the oxidizing substrate of *M*. *tuberculosis* PrxQ B [30]. Peroxynitrite was formed *in vivo* from the diffusion-controlled reaction between NO and O_2_•−, and has been implicated in promoting the development of various pathologies [53]. Moreover, the amount of peroxynitrite-induced damage was not inferior to that by the other peroxides [53]. Thus, the ability of *C*. *glutamicum* PrxQ to eliminate peroxynitrite was detected. After PrxQ (15 μM) was mixed with peroxynitrite (15 μM), peroxynitrite was quickly reduced, confirming its peroxynitrite reductase ability (S3 Fig). However, peroxynitrite is extremely unstable and decays mostly to nitrate with a half life of 0.3 s^-1^at physiological pH or of 0.078 s^-1^ at pH 8. Therefore, the capacity of PrxQ to reduce peroxynitrite was analyzed by competition approaches as described but not an NADPH-coupled spectrophotometric method [35–39]. As shown in Fig 4C, the experiment that HRP (5 μM) was rapidly mixed with peroxynitrite (1 μM) in the absence of PrxQ resulted in the stoichiometric formation of HRP-Compound I, while addition of PrxQ obviously inhibited the HRP-compound I formation and increased the observed rate constant of HRP compound I formation (Fig 4C). The slope of the plot of *k*_obs_ vs PrxQ concentration showed that the rate constant of the reduction of peroxynitrite by PrxQ was of (1.3 ± 0.4)×10^6^ M^−1^ · s^−1^ at pH 7.4 and 25°C, similar to the result of Reyes et al. reported for *M*. *tuberculosis* PrxQ B (1.4 ± 0.3 ×10^6^ M^−1^ · s^−1^ at pH 7.4 and 25°C)[30]. Together, the preferred substrate of *C*. *glutamicum* PrxQ is CHP and peroxynitrite, followed by H_2_O_2_, but *t*-BOOH is the poorest substrate in all cases.

### Enzymatic properties of the PrxQ variants

**Fig 5:**
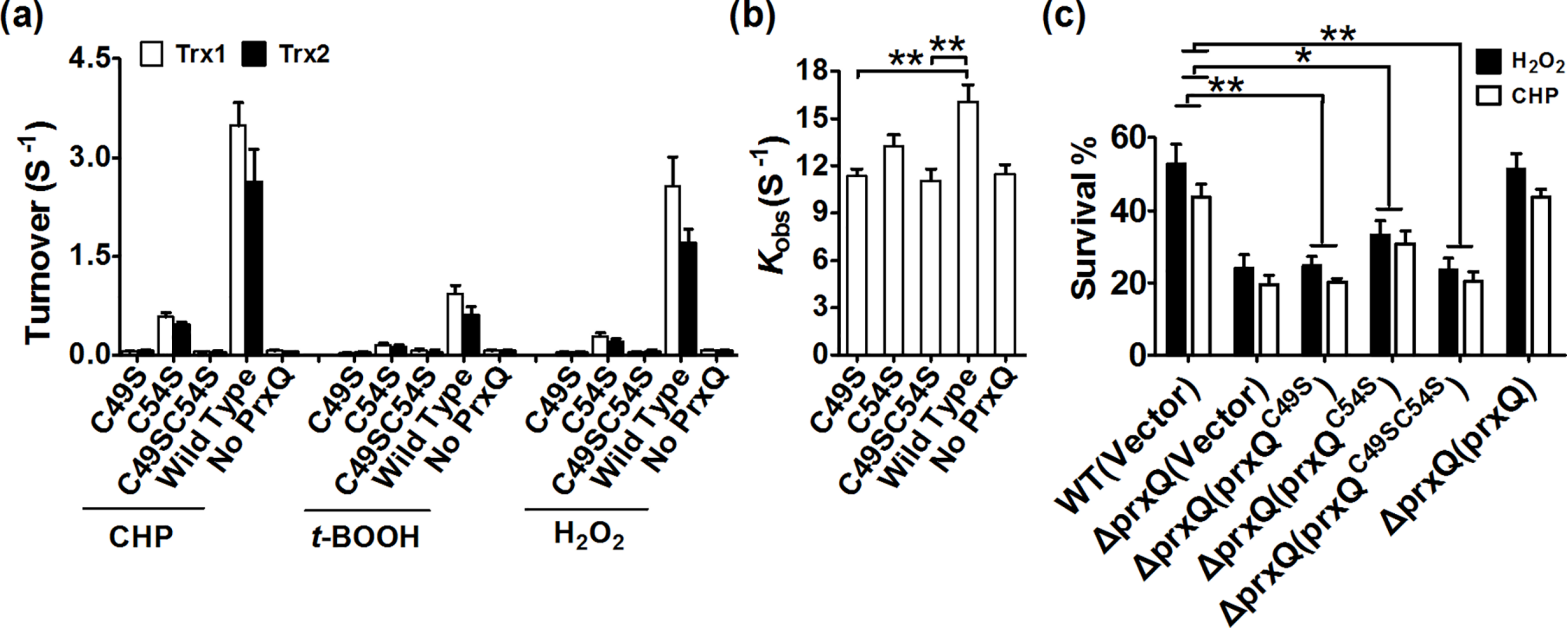
Cys49 and Cys54 are essential amino acids for the peroxidase activity of peroxiredoxin Q (PrxQ). (**a**) Cys49 and Cys54 are required for PrxQ’s peroxidase activity. The assay was performed as in Fig 4A. Mean values with standard deviations (error bars) from at least three repeats are shown. (**b**) Kinetics of peroxynitrite reduction by different PrxQ variants. The assay was performed as in Fig 4B. **: *P*≤0.01. (**c**) PrxQ-mediated protection against diverse stresses is dependent on the active-site cysteines of Cys49 and Cys54. The *prxQ* mutants were transformed with pXMJ19-*prxQ*, pXMJ19-*prxQ*:*C49S*, pXMJ19-*prxQ*:*C54S*, and pXMJ19-*prxQ*:*C49SC54S*, and tested for sensitivity to CHP and H_2_O_2_ in terms of their survival rate, as described in Fig 2A. Mean values with standard deviations (error bars) from at least three repeats are shown. **: *P*≤0.01; *: *P*≤0.05.

Amino acid sequence showed that PrxQ contains two conserved Cys residues at positions 49 and 54 (S1 Fig). In order to determine whether *C*. *glutamicum* PrxQ acts through a 1-Cys or 2-Cys mechanism, the peroxidase activities of single mutated forms of *C*. *glutamicum* PrxQ in each of its two Cys residues (two single-substitution mutations of Cys to Ser, i.e. PrxQ:C49S and PrxQ:C54S) and the double variants PrxQ:C49SC54S were assayed. As shown in Fig 5A, mutant of Cys49 led to a complete loss of the ability of PrxQ to detoxify peroxides, and peroxidase activity was substantially all lost when PrxQ Cys^54^ was replaced with Ser, indicating Cys49 and Cys54 had a very crucial role in catalysis process. Thus, it is concluded that Trx can efficiently reduce PrxQ containing Cys49 and Cys54 under oxidative stress, while Trx is not able to effectively reduce 1-Cys PrxQ variant (PrxQ:C49S and PrxQ:C54S) (Fig 5A).

These findings promoted us to compare the ability of PrxQ variants *in vivo* to complement the oxidation-sensitive phenotype of the Δ*prxQ* mutant (Fig 5B). While the wild-type *prxQ* gene completely efficiently recovered the sensitivity of the *prxQ* mutant to H_2_O_2_, CHP, CdCl_2_, and NiSO_4_, complementation of the *prxQ*:C49S mutant genes had marginal effects on recovery of the survival rates of the Δ*prxQ* mutant under adverse stress conditions and Δ*prxQ*(*prxQ*:C54S) strains only mildly enhanced such tolerance (Fig 5B). These findings indicate that, *in vivo*, the Cys49 and Cys54 residues of PrxQ played an antioxidant role upon exposure to different kinds of oxidative stress.

### Cys49 and Cys54 are the peroxidative cysteine that is oxidized to sulfenic acid and the resolving Cys residue, respectively

**Fig 6:**
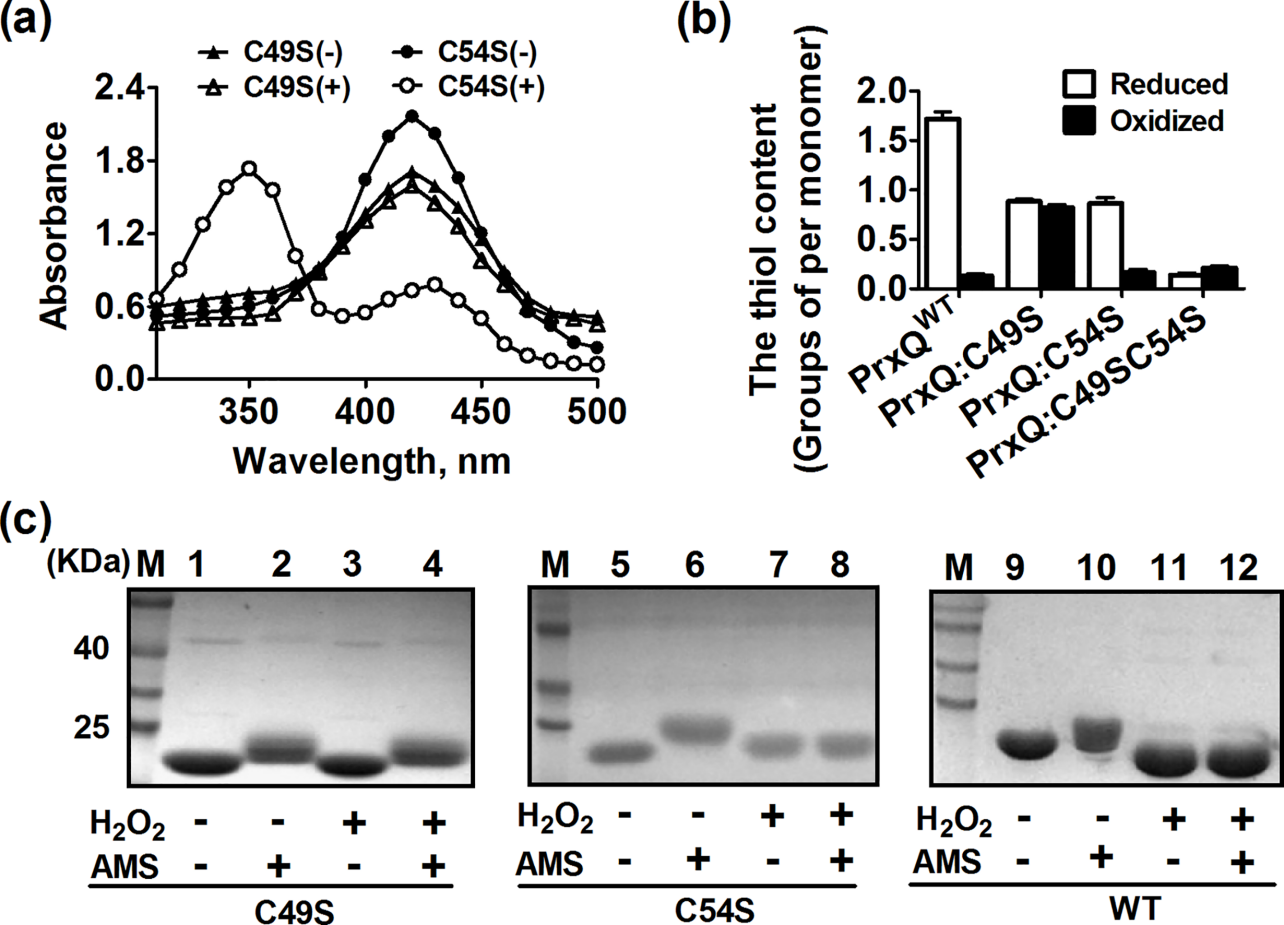
Cys49 and Cys54 are the peroxidative cysteine (C_P_) and the resolving cysteine (C_R_), respectively. (**a**) Spectrophotometric analysis of NBD-labeled PrxQ variants. Proteins treated with or without H_2_O_2_ were modified with NBD-Cl. The resulting proteins were analyzed spectrophotometrically at 200 to 600 nm. (**b**) Quantification of free PrxQ thiol levels in reduced and oxidized proteins. H_2_O_2_- and DTT-treated proteins (10 μM) were admixed 2 mM with 5, 5’-dithio-bis(2-nitrobenzoic acid) (DTNB) in 50 mM Tris-HCl buffer (pH 8.0), and the absorbance was monitored at 412 nm against a 2 mM DTNB solution as reference. These data are means of the values obtained from three independent assays. (**c**) Oxidative states of cysteine residues in PrxQ variants were examined. 50 mM DTT-treated proteins (25 μM) were cultivated with (+) or without (-) 2.5 mM H_2_O_2_, and then free thiol groups were modified with AMS. The modified samples were separated on 15% non-reducing SDS-PAGE.

Amino acid sequence comparison showed that Cys49 is the peroxidative cysteine residue (C_P_) that is homologous to catalytic Cys present in all selected PrxQ containing a P-(X)_3_-(T/S)-[P/F]-(X)-C_P_-[T/S/P] motif that had been reported to be involved in catalysis via the transient formation of a sulfenic acid [30]. However, Cys54 might be the resolving Cys residues (C_R_), which was because it located five residues downstream from the C_P_. Therefore, to investigate whether Cys49 and Cys54 have the above speculative function, variants PrxQ:C49S and PrxQ:C54S with and without previous exposure to H_2_O_2_ were used, which were then treated with NBD-Cl. NBD-Cl can exclusively react with thiol groups and sulfenic acids, and the covalent attachment of NBD-Cl generated an absorption peak at about 420 nm upon reaction with thiol groups, whereas it peaked at about 347 nm upon reaction with sulfenic acids [42]. Following the reaction with NBD-Cl, the absorption spectrum of the PrxQ:C49S variant was unchanged before and after exposure to CHP (Fig 6A), exhibiting only the peak at 420 nm. The PrxQ:C54S variant with H_2_O_2_ treatment showed soret bands at 347 nm and 420 nm, indicating the simultaneous reaction of NBD-Cl with sulfenic acids and free thiol groups. Similarly, Cys oxidation was also quantified by measuring the free thiol content per PrxQ monomer by 5, 5-dithiobis (2-nitrobenoic acid) (DTNB) assay. As expected, the PrxQ:C49S variant showed one thiol per monomer before and after H_2_O_2_ treatment. However, the PrxQ:C54S under H_2_O_2_ treatment lost one thiol groups, compared to the thiol content of DTT-treated states (Fig 6B). TThe redox state of thiol was further examined using AMS covalent modification. Since the molecular mass of AMS is 0.5 KD and covalent modification of AMS is irreversible, the apparent electrophoretic mobility delay would emerge in proportion to the amount of free thiol groups [43]. The H_2_O_2_-treated, AMS-modified PrxQ:C54S migrated as same as the H_2_O_2_-treated, AMS-unmodified states and faster than its H_2_O_2_-untreated, AMS-modified form (Fig 6C), indicating that Cys49 was not existed in thiols in the H_2_O_2_-treated PrxQ:C54S variant (Fig 6C, lanes 7 and 8). These results suggest that Cys49 is the peroxidatic Cys, which is more susceptible to oxidation upon exposure to H_2_O_2_.

However, H_2_O_2_-treated, AMS-modified PrxQ:C49S migrated slower compared to that of H_2_O_2_-treated, AMS-unmodified form and the same as that of H_2_O_2_-untreated, AMS-modified form (Fig 6C, lanes 2–4), indicating that the Cys54 was still in thiol form under exposure to H_2_O_2_. NBD-Cl assay also revealed that the absorption spectra of the PrxQ:C49S proteins were unvaried before or after exposure to H_2_O_2_ (Fig 6A). In line with the above phenomena, the thiol content of DTT-treated states was 0.93 ± 0.09 thiol groups per monomer, while the H_2_O_2_-treated PrxQ:C49S proteins lost zero thiol group compared to its reduced states (Fig 6B). These data indicate that the PrxQ:C49S variants are not oxidized under H_2_O_2_ treatment and Cys54 still remain be the thiol state.

### Cys49 and Cys54 form an intramolecular disulfide bond under oxidative stress

To test if these two Cys could undergo disulfide bonding after oxidation, AMS modification and the free thiol content assay were used. As shown in Fig 6C, PrxQ WT treated with H_2_O_2_ was not modified by AMS, because AMS and H_2_O_2_-treated PrxQ WT migrated more rapidly than that of AMS-modified, H_2_O_2_-untreated form, and the same as that of H_2_O_2_-treated, AMS-unmodified form. The result was further confirmed by measuring the thiol content with the DTNB assay (Fig 6B). The DTT-treated PrxQ WT contained 1.74 ± 0.13 thiol groups per monomer, but the thiol content decreased to 0.16 ± 0.03 when PrxQ WT was treated with H_2_O_2_. The difference of 1.56 thiol groups between the two preparations is linked to the fully oxidation of PrxQ WT after H_2_O_2_ treatment. These data indicate that PrxQ WT is fully oxidized by H_2_O_2_ to form disulfide bond between Cys49 and Cys54.

### PrxQ forms a mixed disulfide with Trx

**Fig 7:**
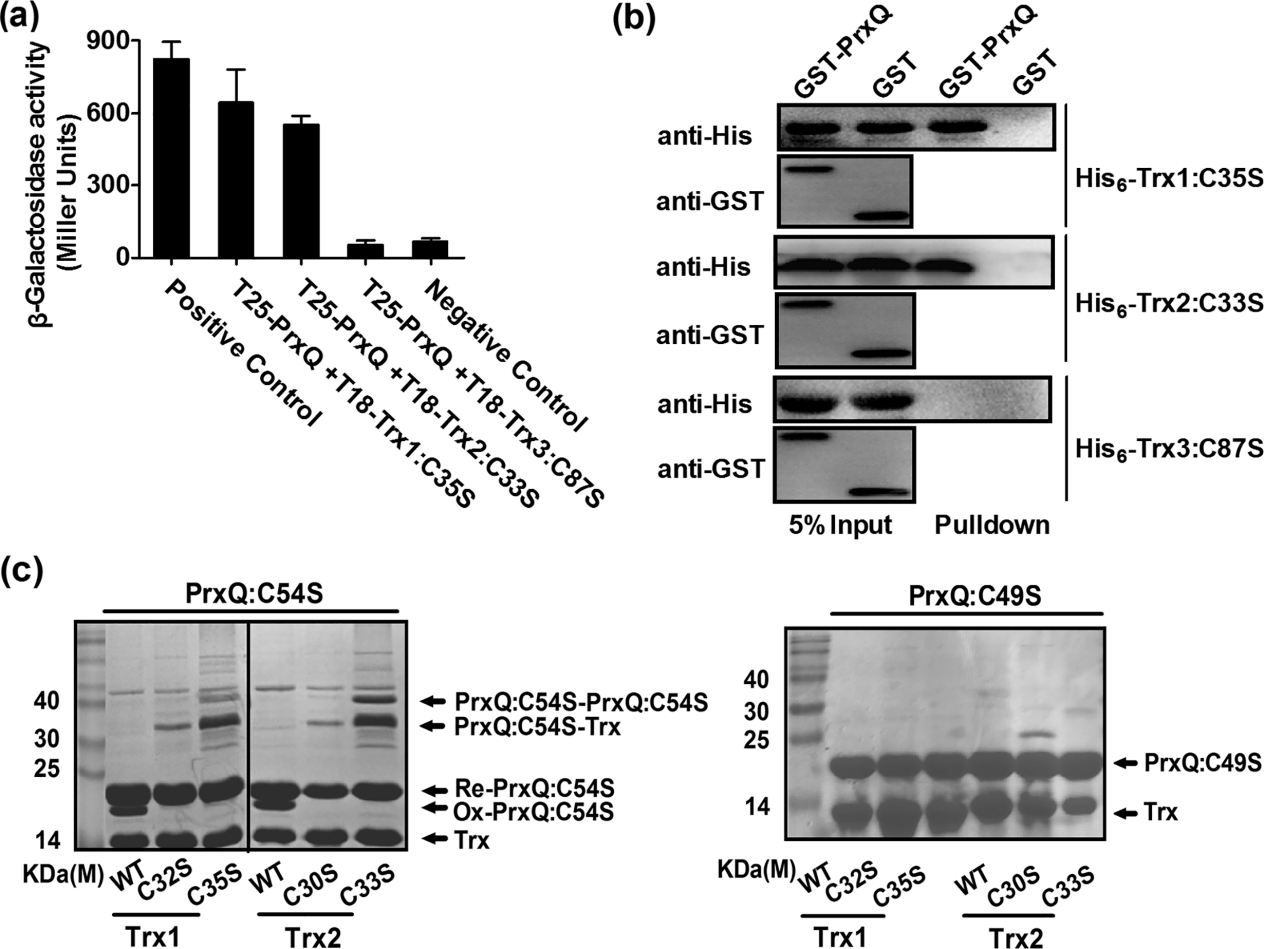
Peroxiredoxin Q (PrxQ) formed a mixed disulfide with thioredoxin (Trx). (**a**) Bacterial two-hybrid complementation assays were carried out to analyze the interactions between PrxQ and Trx. Assays were performed in triplicate. Mean values with standard deviations (error bars) are shown. (**b**) His_6_-Trx1:C35S or His_6_-Trx2:C33S was retained by agarose beads coated with GST-PrxQ. GST-Bind beads coated with GST- PrxQ or GST were incubated with His_6_-Trx1:C35S or His_6_-Trx2:C33S and protein complexes specifically retained by GST- PrxQ-coated beads were detected with anti-His antibody. (**c**) Sulfenic acid-containing PrxQ created stable association with Trx1:C35S/Trx2:C33S. Non-reducing 15% SDS-PAGE showed PrxQ:C49S and PrxQ:C54S incubated with each Trx1 (WT, Trx1:C32S and Trx1:C35S) or each Trx2 (WT, Trx2:C30S and Trx:C33S) in the presence of 50 μM H_2_O_2_. PrxQ:C54S_ox_ and Prx:C54S_red_ expressed oxidized and reduced PrxQ:C54S, respectively.

To further confirm the results that PrxQ uses Trx as a reducing power, we investigated the direct interaction between Trx1/Trx2 and PrxQ by glutathione S-transferase (GST) pull-down and bacterial two-hybrid assays. Early reports suggested that sulfhydryl group regeneration at the active-site Cys of Prx was performed by the N-terminal Cys of Trx at the CXXC active sites (Trx1_C^32^XXC^35^ and Trx2_C^30^XXC^33^), and a transient intermolecular disulfide bond linking Prx with Trx can be stabilized by removing an internal cysteine of Trx at the CXXC active sites [54–55]. Therefore, to avoid attacking by the C-terminal Cys of Trx at the CXXC active sites, single mutants of Trx protein, namely, Trx1:C35S and Trx2:C33S, were constructed. As shown in Fig 7A, the Trx1:C35S and Trx2:C33S mutants showed a very strong interaction with PrxQ, similar to the positive control. To verify the Trx-PrxQ interaction seen in the bacterial two-hybrid assay, we produced recombinant His_6_-Trx:CXXS/GST-PrxQ and tested their interactions with GST-PrxQ using the GST pull-down assay *in vitro*. As shown in Fig 7B, we observed the specific interaction between GST-PrxQ and His_6_-Trx1:C35S/His_6_-Trx2:C33S.

At the stage of PrxQ regeneration, the formation of a transient intermolecular disulfide bond between PrxQ and Trx1-Cys^32^/Trx2-Cys^30^ was identified (Fig 7A and 7B). However, it remained unclear which cysteine of PrxQ (Cys-49 or Cys-54) is favored by Trx. To clarify this, two single variants PrxQ:C49S and PrxQ:C54S were incubated with three versions of Trx [Trx1 WT (wild type), Trx1:C32S, and Trx1:C35S; Trx2 WT(wild type), Trx2:C30S, and Trx2:C33S] in the presence of H_2_O_2_. Clear polypeptide complexes occurred on non-reducing SDS-PAGE when mixing PrxQ:C54S and Trx1:C35S/Trx2:C33S in the presence of H_2_O_2_ (Fig 7C, lanes 3 and 6), which did not exist in only H_2_O_2_-treated Trx1:C35S/Trx2:C32S/PrxQ:C54S (S4 Fig) and the mixture of Trx1:C35S/Trx2:C32S and PrxQ:C54S under H_2_O_2_ treatment separated on reducing SDS-PAGE (S5 Fig).The size of the heterodimer polypeptide (approximately 35kD) was in good agreement with the addition of one Trx to one PrxQ. Unexpectedly, formation of the new polymer complexes (about 40 KDa) was also observed in the H_2_O_2_-treated PrxQ:C54S variant, though to a much lesser extent. These results suggest that Cys49 was involved in formation of nonspecifically dimers through intermolecular disulfide bonds. Although the mechanisms remain unrevealed, the formation of the nonspecific dimers upon strong oxidative stress treatment has also been reported for the thiol-dependent peroxidase Ohr [56] and AphC [57]. The observation that WT Trx did not form stable heterodimers with PrxQ:C54S was in line with the formation of a transient intermolecular disulfide bridge that is subsequently reduced by the second active site Cys of Trx. In addition, the various Trxs were unable to form dimers with PrxQ:C49S (Fig 7C). These results suggest that Cys-49 of PrxQ is favored by Trx1-Cys^32^/Trx2-Cys^30^. The above *in vitro* results indicate that PrxQ can indeed be regenerated by Trx.

### Oxidant-induced *prxQ* expression and its positive regulation by SigH

**Fig 8:**
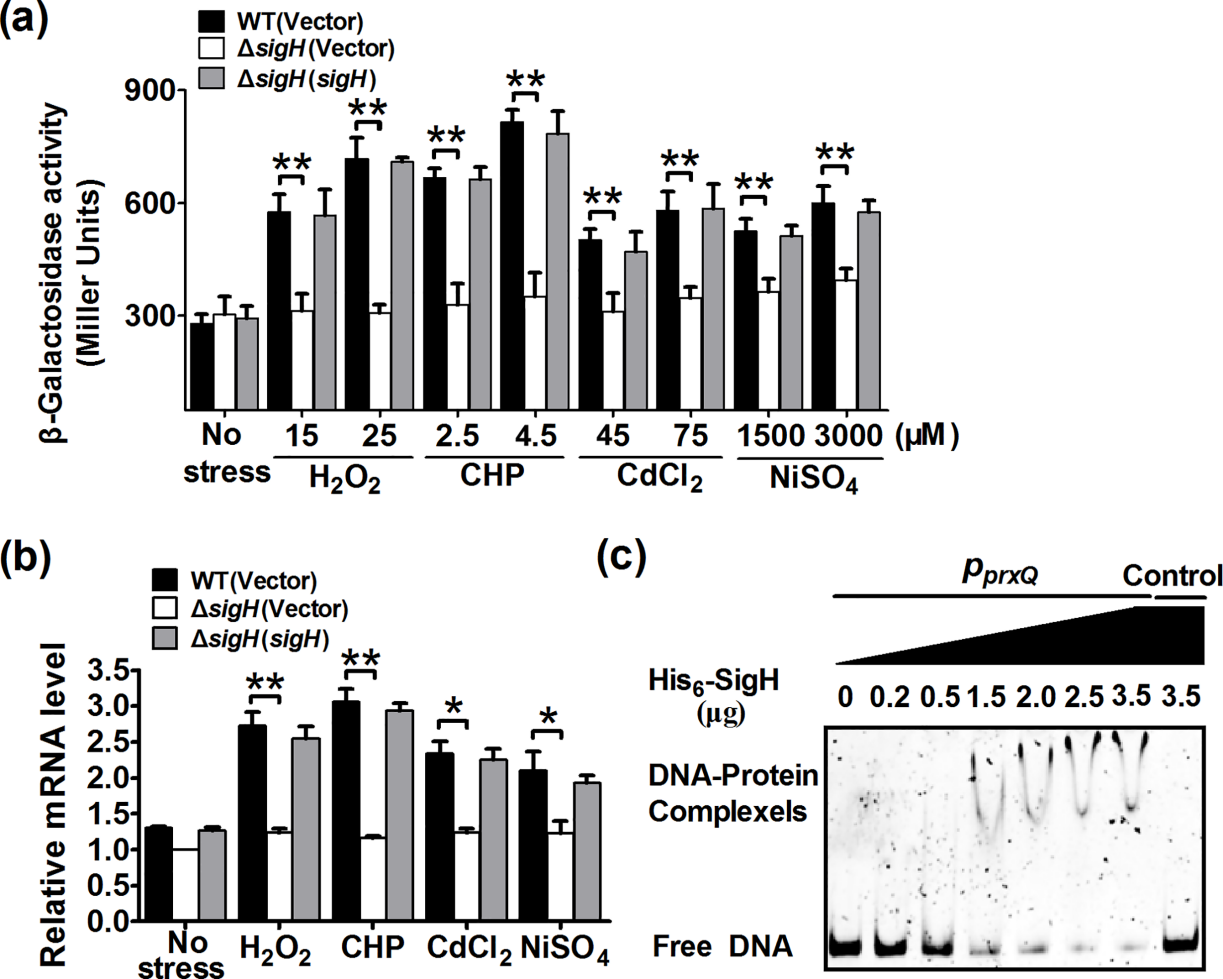
Positive regulation of *C*. *glutamicum prxQ* expression by SigH. **(a)** β-Galactosidase analysis of the *prxQ* promoter activity by using the transcriptional *P*_*prxQ*_::*lacZ* chromosomal fusion reporter expressed in indicated strains under different adverse condition for 30 min. β-Galactosidase activity was examined as described in “Materials and Methods”. Mean values with standard deviations (error bar) from at least three repeats are shown. **, *P≤*0.01; *, *P≤*0.05. **(b)** Quantitative RT-PCR analyses of *prxQ* expression in indicated strains under adverse stress conditions. Exponentially growing relevant *C*. *glutamicum* strains were exposed to different adverse stresses at indicated concentrations for 30 min. The expression levels were measured by qRT-PCR. The mRNA levels are presented relative to the value obtained from wild-type cells without treatment. Relative transcript levels of the gene without treatment were set at a value of 1.0.The values represent the mean results from three independent cultivations, with standard errors. **: *P≤*0.01; *, *P≤*0.05. **(c)** Binding of SigH to the *prxQ* promoter examined by EMSA. The increasing amounts of SigH used were 0, 0.2, 0.5, 1.5, 2.0, 2.5, and 3.5 μg (lane1, 2, 3, 4, 5, 6, and 7, respectively). A 300 bp fragment from the *prxQ* coding region was added in the binding assay to determine the binding specificity of His_6_-SigH (lane 8).

As PrxQ is involved in eliminating cellular ROS induced by adverse stresses, *prxQ* expressions in response to adverse stresses were investigated by chromosomal *P*_*prxQ*_::*lacZ* fusion reporter and qRT-PCR analysis. The *lac*Z activity of *P*_*prxQ*_::*lacZ* chromosomal promoter fusion reporter in the RES167 wild type strain was quantitatively measured under H_2_O_2_, CHP, CdCl_2_, and NiSO_4_ at different concentrations (Fig 8A). Concentrations of adverse stress applied were able to reduce the growth rate but under sub-lethal concentrations (S6 Fig). Compared to the untreated samples, the promoter activity of *prxQ* was approximately 2.57-, 2.92-, 2.0-, and 2.15-fold in the wild type RES167 reporter strain treated with H_2_O_2_ (25 mM), CHP (5.5 mM), CdCl_2_ (75 μM), and NiSO_4_ (3 mM), respectively (Fig 8A). Further, expression of the *P*_*prxQ*_::*lac*Z fusion displayed a dose-dependent increase in response to these adverse environmental conditions (Fig 8A). A similar dose-dependent pattern of *prxQ* expression in response to H_2_O_2_, CHP, CdCl_2_, and NiSO_4_ was also observed in qRT-PCR analysis (Fig 8B). The strong induction of *prxQ* by adverse stress suggests that a stress sensor/transcriptional regulator might be involved in the regulation of its expression. As SigH, the stress-responsive extracytoplasmic function-sigma (ECF-σ) factor, was reported to respond to thiol-oxidative stress and regulate the expression of multiple resistance genes [48, 58], we examined whether *prxQ* expression was subjected to SigH regulation by measuring the transcription of chromosomal *P*_*prxQ*_::*lac*Z fusions. Under exposure to H_2_O_2_ (25 mM), CHP (4.5 mM), CdCl_2_ (75 μM), and NiSO_4_ (3 mM) for 30 min, a marked decrease of *prxQ* promoter activity was detected in the Δ*sigH* mutant compared to the wild-type strain (Fig 8A). However, the *prxQ* expression in the Δ*sigH* mutant was almost fully recovered when the regulatory protein was complemented either under adverse stress-treated or -untreated conditions (Fig 8A). A similar pattern of *prxQ* expression in response to various adverse stresses was observed in the qRT-PCR analysis (Fig 8B). The SigH-depdendent *prxQ* expression was further confirmed with *in vitro* EMSA assay by determining the direct interaction between His_6_-SigH and *prxQ* promoter region. Incubation of a 300 bp PCR fragment probe containing the *prxQ* promoter sequence with His_6_-SigH led to the formation of DNA-protein complexes and retard mobility of the probe (Fig 8C), and the substitution of objective probe by a 300 bp control DNA amplified from the *prxQ* coding region abolished the formation of the protein-DNA complex (Fig 8C, lane 8). Furthermore, the DNA-protein complexes increased in response to more His_6_-SigH used in the reactions. Collectively, these results indicate that SigH activates *prxQ* expression by directly binding to the *prxQ* promoter.

## Discussion

Prxs are Cys-based peroxidases that react very rapidly with H_2_O_2_, organic peroxides, and peroxynitrite. According to the number of conserved cysteine residues directly involved in catalysis, Prxs are classified into three types and six subgroups: typical 2-Cys (Prx1), atypical 2-Cys (Tpx, PrxQ, and Prx5), and 1-Cys Prxs (Prx6 and AhpE) [9–11]. Although Prxs have been extensively studied in many bacteria, especially Prx1 and Tpx, less is known about the PrxQ in *C*. *glutamicum*. Here, we present data concerning the molecular mechanism, substrate and donor specificity, and *in vivo* function of PrxQ from *C*. *glutamicum*, which is annotated as the putative PrxQ-BCP subfamily. As expected, *C*. *glutamicum* PrxQ acted as a Trx-dependent atypical prokaryotic 2-Cys Prx, Cys49 and Cys54 of whose was C_P_ and C_R_, respectively. The result was supported by following several lines of evidence: 1) *C*. *glutamicum* PrxQ exclusively received electrons from the Trx/TrxR system to reduce peroxides but not the Mrx1/MSH/Mtr system (Fig 3); 2) The Cys49 is completely essential for activity, whereas the catalytic activity of C54S variant substantially all lost (Fig 5); 3) The thiol content, AMS assay, and non-reducing SDS-PAGE mobility ratio supported the presence of a disulfide bridge between Cys49 and Cys54, because of the introduction of the Ser residue in place of Cys49 or Cys54 leading to be not form disulfide bond between Cys49 and Cys54. We propose here that in the WT form of the enzyme, the regeneration of Cys49 involves the transient formation of a disulfide with Cys54, which is subsequently reduced by Trx (Fig 6); 4) The Cys49 was the peroxidatic cysteine (C_p_), forming a covalent NBD adduct and being not the free thiol group as proven by an absorbance maximum at 347 nm and DTNB analysis when PrxQ:C54S was exposed to H_2_O_2_ (Fig 6); 5) *C*. *glutamicum* PrxQ was indeed monomeric, which was verified by gel filtration chromatography and non-reducing SDS-PAGE (Fig 1); 6) The Cys49 was a necessary residue to the Trx/TrxR pathway, being residues of interaction between PrxQ and Trx. The absence of heterodimer formation was observed in the mixture of the PrxQ:C49S and Trx1:C35S/Trx2:C33S whereas the heterodimer occurred in the reaction with the PrxQ:C53S and Trx1:C35S/Trx2:C33S (Fig 7).

From the biochemical analysis of *C*. *glutamicum* PrxQ, we found that *C*. *glutamicum* PrxQ was able to reduce a broad spectrum of hydroperoxides including H_2_O_2_, *t*-BOOH, CHP and peroxynitrite (Fig 4), but kinetic measurements indicated a preference for peroxynitrite and CHP, while reduction of *t*-BOOH was much slower. This is consistent to the finding that *M*. *tuberculosis* PrxQ B had various oxidizing substrates [30]. However, *M*. *tuberculosis* PrxQ A lacked the capacity of reducing peroxynitrite [30]. It is shown that *C*. *glutamicum* PrxQ had high identities (49%) with *M*. *tuberculosis* PrxQ B, while no significant correlation with *M*. *tuberculosis* PrxQ A (S1 Fig). Therefore, we suggested that the different behavior of these kinds of PrxQ could be explained based on their structure difference. More notably, the catalytic efficiency of *C*. *glutamicum* PrxQ for H_2_O_2_ (*k*_cat_/*K*_m_ of about 4.2~9.6 ×10^3^ M^-1^·S^-1^) is lower compared to that of CHP and peroxynitrite, while it is comparable with the value obtained for the *E*. *coli* BCP and Poplar PrxQ (2.45×10^3^ M^-1^·S^-1^ and 7.98×10^3^ M^-1^·S^-1^) and to those of the 2-Cys Prx from *Arabidopsis* (36×10^3^ M^-1^· S^-1^) [17, 29, 59]. Given the important roles postulated for H_2_O_2_, especially in the gene transcription, cellular signal transduction, bactericidal action of neutrophils and macrophages, these findings are likely to be of physiological importance that was the significance of PrxQ as a means to mediated H_2_O_2_-regulated normal biological functions.

PrxQ was reported to be regenerated by the classic Trx/TrxR reducing system [17, 29–30]. In the present study, we confirmed that *C*. *glutamicum* PrxQ can also be efficiently reduced by the Trx/TrxR system (Fig 3), suggesting that the Trx/TrxR-based reducing system is a common reducing system for PrxQ in different organisms. It was important to mention that *C*. *glutamicum* possesses three thioredoxin encoding genes. Therefore, we have carried out an extensive study of the electron donor specificity of PrxQ, testing a large number of Trx. Results showed that their efficiencies differed. The most efficient reductant was Trx1, similar to the reported result of Poplar PrxQ [29]. Actually, Poplar type II Prx, having the ability of interacting with Grx, has two Glu residues equivalent to the Asp residues in position 148 and 156, relating to the interaction between Prx and Grx [51, 60]. When comparing *C*. *glutamicum* PrxQ with poplar type II Prx, we found that *C*. *glutamicum* PrxQ had also two Glu residues (at positions 149 and 154) in positions equivalent to Asp148 and 154 of the Prx/Grx fusion. Thus, the possibility that Mrx1/MSH/Mtr system, considered as functional equivalent to the GSH/Grx/GR system, donates electrons to PrxQ via similar Prx/Grx interaction way cannot be excluded in *C*. *glutamicum*. However, we found that regeneration of *C*. *glutamicum* PrxQ was not supported by the Mrx1/Mtr/MSH reducing system (S2 Fig). Consistently, the Grx/GSH/GR system failed to support PrxQ activity in Poplar [29] and confirmed in *C*. *glutamicum* in the present study. We suggested that the finding was related to the 2-Cys mechanism of the Trx/TrxR system but independent of 1-Cys mechanism (i.e. the mycothiolation/demycothiolation reduction mechanism) of the Mrx1/MSH/Mtr system [28, 61]. MSH reduces the sulfenic acid intermediate of peroxidase via the formation of an *S*-mycothiolated peroxidase intermediate (Protein-SSM) which is then recycled by Mrx1 and the second molecule of MSH. More notably, an interesting phenomenon that PrxQ:C54S had mild effects on recovery of the survival rates of the Δ*prxQ* mutant under adverse stress conditions but not active *in vitro* with the Trx/TrxR system as electron donor was observed (Fig 5). The reason why PrxQ:C54S has different behaviors *in vivo* and *in vitro* is possibly due to the mechanism employed in reducing PrxQ:C54S. It is shown that upon H_2_O_2_ treatment, a stable sulfenic acid intermediate occurred in Cys49 of PrxQ:C54S variant (Fig 6). Moreover, the sulfenic acid formed in C_P_ of C_R_-lacked PrxQ is more efficiently reduced by Grx/GSH/GR system [18]. This could be an indication that the Cys-SOH derivative of PrxQ:C54S was regenerated by the Mrx1/MSH/Mtr sysem but not by the Trx/TrxR system under oxidative stress. The physiological and biochemical mechanisms of the phenomenon need to be further investigated in the future.

Although it has long been known that Prx is involved in physiological and biochemical functions in numerous bacteria, PrxQ in *C*. *glutamicum* remained uncharacterized [4–5]. According to a cell viability assay, when the WT and Δ*prxQ* strains were exposed to different kinds of stressors, the former was more sensitive than the latter and the hypersensitive phenotype could be completely reversed by complementation with the *prx*Q gene (Fig 2). This means that, to some extent, PrxQ helps bacteria endure diverse stressors. Numerous studies have described that the exposure of microorganisms to various stressors, such as heavy metals, antibiotics, alkylating agents, and acids, can increase the production of ROS and then induce oxidative stress [1]. Reports have also stated that Prx can remove intracellular ROS, thereby decreasing the amount of damage suffered by bacteria [5]. Against this background, we examined the intracellular ROS level and found that, under various stressors, the levels markedly increased, especially in the Δ*prxQ* mutant. In addition, previous studies have demonstrated that ROS accumulation results in the irreversible formation of sulfoxidation products, eventually leading to protein carbonylation [51–52]. Therefore, in this study, carbonyl assays were performed on total proteins isolated from Δ*prxQ* and WT strains. After exposure to different stresses, the carbonylation level of protein extracts was significantly lower in the WT strain than in the Δ*prxQ* strain (Fig 2). The physiological roles of PrxQ in resistance to multiple oxidative stresses were also corroborated by the induced expression of *prxQ* in *C*. *glutamicum* under various oxidative stresses (Fig 8). These results not only strongly suggest that *prxQ* expression may be important for bacteria growth under oxidative stress conditions, but also provide a promising strategy to engineer robust industrial strains in the future.

**Fig 9:**
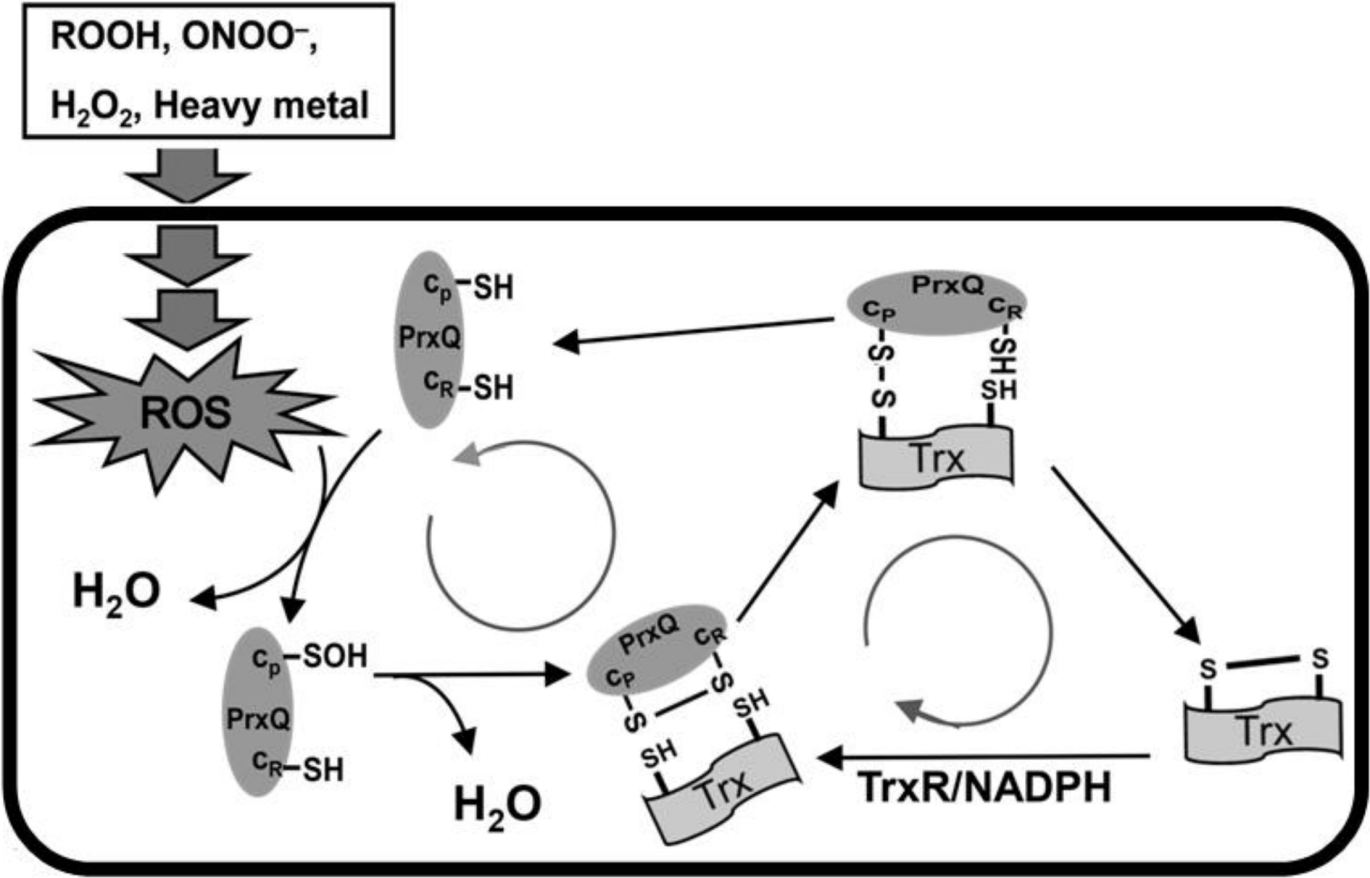
Model of resistance to diverse stresses in *Corynebacterium glutamicum* based on peroxiredoxin Q/thioredoxin (PrxQ/Trx). The first step consists of H_2_O_2_ reduction with the concomitant formation of a stable sulfenic acid intermediate on catalytic Cys49 of PrxQ. Nucleophilic attack by Cys54 on the sulfenic acid intermediate leads to the release of one molecule of H_2_O and the formation of a transient disulfide bond between Cys49 and Cys54. This disulfide bond is reduced by the Trx/thioreductase recycling system to reform the reduced peroxidatic Cys. Then, the regenerated PrxQ is ready for another catalytic cycle.

Based on our experimental data, a catalytic model for the alternatively reduction of PrxQ by Trx would be hypothesized (Fig 9). The first step consists of H_2_O_2_ reduction with the concomitant formation of a stable sulfenic acid intermediate on catalytic Cys49 of PrxQ. Nucleophilic attack by Cys54 on the sulfenic acid intermediate leads to the release of one molecule of H_2_O and the formation of a transient disulfide bond between Cys49 and Cys54. This disulfide bond is reduced by the Trx/TrxR recycling system to reform the reduced peroxidatic Cys. Then, the regenerated PrxQ is ready for another catalytic cycle.

## Supporting information

S1 Table Bacterial strains, plasmids, and primers used in this study. (DOCX)

S2 Table Primers used in this study. (DOCX)

S3 Table Trx-dependent avtivities of PrxQ towards different substrates. (DOCX)

S1 Fig Multiple sequence alignment of PrxQ with other representative PrxQs. The lack background showed the strong conserved Cys in positions 49 and 53 (*C*. *glutamicum* PrxQ numbering). Accession numbers: *C*. *glutamicum* ATCC 13032 (NP_601690); *E*. *coli* (P0AE52); poplar (AY530803); *Sedum lineare* (BAA90524); *Arabidopsis thaliana* (BAB01069); *accharomyces cerevisiae* (CAA86239); *Nostoc sp*. *PCC 7120* (BAB74202); *Agrobacterium fabrum str*. *C58* (NP_354814); *Rhizobium etli* (WP_011425522); *Synechococcus* (WP_011129017); *Mycobacterium tuberculosis* (NP_216124); *Mycobacterium tuberculosis* (NP_216641).
(TIF)

S2 Fig Reduction of various peroxides by PrxQ coupled to the Mrx1/Mtr/MSH regeneration pathway. The reduction of peroxides was recorded by measuring the decrease of NADPH oxidation at 340 nm. The reactions omitted the Mrx1 electron pathway, PrxQ, or peroxide served as negative controls. Similar results were obtained in three independent experiments, and data shown are from one representative experiment done in triplicate.
(TIF)

S3 Fig Peroxynitrite reductase activity of PrxQ. Time trace of peroxynitrite decay in absence and presence of PrxQ B (15 μM) at pH 7.4 at 25°C. The data were presented as means of the values obtained from three independent assays.
(TIF)

S4 Fig Redox response of proteins detected by non-reducing SDS-PAGE. Non-reducing 15% SDS-PAGE showing states of PrxQ (Its variants), Trx1 (WT and its variants) and Trx2 (WT and its variants) (20 μM) in the presence of 50 μM H_2_O_2_.
(TIF)

S5 Fig The mixtures of PrxQ:C49S/PrxQ:C54S and Trx1(WT and its variants) or Trx2(WT and its variants) were detected on reducing SDS-PAGE. M: protein molecular weight marker.
(TIF)

S6 Fig Survival rates of *C*. *glutamicum* in response to sub-lethal concentrations of different peroxides. *C*. *glutamicum* wild type was grown in LB medium to an OD_600_ of 1.0 and exposed to different concentrations of various peroxides at 30°C for 30 min. After treatment, the cultures were serially diluted, spreaded on LB plates and incubated at 30°C for 36 h. Survival percentages were calculated as [(CFU ml^-1^ with stress)/(CFU ml^-1^ without stress)]×100. Mean values with standard deviations (error bars) from at least three repeats are shown.
(TIF)
