# Supplementary material for: A thioredoxin-dependent peroxiredoxin Q from Corynebacterium glutamicum plays an important role in defense against oxidative stress
Source: PLoS One. 2018 Feb 13;13(2):e0192674. doi: 10.1371/journal.pone.0192674 (PMC5811025; doi:10.1371/journal.pone.0192674)
Supplement: S1 Table — (DOCX) [file pone.0192674.s001.docx]

**S1 Table. Bacterial strains and plasmids used in this study.**

| **Strains or plasmids** | **Relevant genotype description** | **References** | |
| --- | --- | --- | --- |
| **Strains** | | | |
| ***Corynebacterium glutamicum*** | | | |
| RES167 | Restriction-deficient mutant of ATCC13032, Δ(*cglIM-cglIR-cglIIR*) | | [1] |
| Δ*prxQ* | *prxQ* deleted in RES167 | | This study |
| WT(Vector) | wild type RES167 containing pXMJ19 vector | | [2] |
| Δ*prxQ*(*prxQ*) | Complementation of *prxQ* in RES167 | | This study |
| Δ*prxQ*(*prxQ:C49S*) | Complementation of *prxQ:C49S* in RES167 | | This study |
| Δ*prxQ*(*prxQ:C54S*) | Complementation of *prxQ*:*C54S* in RES167 | | This study |
| Δ*prxQ*(*prxQ:C49SC54S*) | Complementation of *prxQ:C49SC54S* in RES167 | | This study |
| Δ*prxQ*(Vector) | Δ*prxQ* containing pXMJ19 vector | | This study |
| Δ*sigH* | *sigH* deleted in RES167 | | [3] |
| ***E. coli*** | | | |
| BL21(DE3) | Host for expression vector pET28a | | Novagen |
| XL1-blue | Host for expression vector pGEX6p-1 | | Stratagene |
| JM109 | *recA1 supE44 endA1 hsdR17 gyrA96 relA1 thi* Δ(*lac-proAB*)F′(*traD36 proABlacI*^q^ *lacΔZM15*) | | Stratagene |
| **Plasmids** | | | |
| pK18*mobsacB* | Suicide plasmid carrying *sacB* for selecting double crossover in *C. glutamicum*, Km^r^ | | [4] |
| pK18*mobsacB-*Δ*prxQ* | Construct used for in-frame deletion of *prxQ* | | This study |
| pK18*mobsacB-P_prxQ_::lacZ* | *P_prxQ_::lacZ* fusion in pK18*mobsacB* | | This study |
| pXMJ19 | Shuttle vector (*Ptac lacI^q^ pBL1 oriV_C. glutamicum_* pK18 *oriV_E. coli_*) | | [5] |
| pXMJ19-*prxQ* | *prxQ* cloned into pXMJ19 for complementation | | This study |
| pXMJ19-*prxQ:C49S* | *prxQ:C49S* cloned into pXMJ19 for complementation | | This study |
| pXMJ19-*prxQ:C54S* | *prxQ:C54S* cloned into pXMJ19 for complementation | | This study |
| pXMJ19-*prxQ:C49SC54S* | *prxQ:C49SC54S* cloned into pXMJ19 for complementation | | This study |
| pXMJ19-*sigH* | *sigH* cloned into pXMJ19 for complementation | | [6] |
| pKT25-zip | BTH positive control | | [6] |
| pUT18C-zip | BTH positive control | | [6] |
| pKT25M | Modified pKT25 | | [3] |
| pUT18CM | Modified pUT18C | | [3] |
| pKT25M-*prxQ* | *prxQ* in pKT25M | | This study |
| pUT18CM-*trx1:C35S* | *trx1:C35S* in pUT18CM | | This study |
| pUT18CM-*trx2:C33S* | *trx2:C33S* in pUT18CM | | This study |
| pGEX6P-1 | Expression vector, T7 promoter, GST tag coding sequence, Amp^r^, pBR322 origin, contains a broad-host-range origin of replication *oriV* | | Amersham Biosciences |
| pGEX6P-1-*prxQ* | *prxQ* in pGEX6P-1 | | This study |
| pET28a | Expression vector with N-terminal hexahistidine affinity tag | | Novagen |
| pET28a*-trx1* | *trx1* in pET28a | | [3] |
| pET28a-*trx2* | *trx2* in pET28a | | This study |
| pET28a-*trx3* | *trx3* in pET28a | | This study |
| pET28a-*trx1:C35S* | *trx1:C35S* mutant in pET28a | | [3] |
| pET28a-*trx1:C32S* | *trx1:C32S* mutant in pET28a | | [3] |
| pET28a-*trx2:C30S* | *Trx2:C33S* mutant in pET28a | | This study |
| pET28a-*trx2:C33S* | *Trx2:C33S* mutant in pET28a | | This study |
| pET28a*-trxR* | *trxR* in pET28a | | [3] |
| pET28a*-prxQ* | *prxQ* in pET28a | | This study |
| pET28a*-prxQ:C49S* | *prxQ:C49S*in pET28a | | This study |
| pET28a-*prxQ:C54S* | *prxQ:C53S* in pET28a | | This study |
| pET28a*-prxQ:C49SC54S* | *prxQ:C49SC53S* in pET28a | | This study |

**References**

1. Tauch A, Kirchner O, Lo¨ffler B, Go¨tker S, Pu¨hler A, Kalinowski J. Efficient electrotransformation of corynebacterium diphtheriae with a mini-replicon derived from the *Corynebacterium glutamicum* plasmid pGA1. [Curr Microbiol](http://www.ncbi.nlm.nih.gov/pubmed/?term=Efficient+electrotransformation+of+corynebacterium+diphtheriae+with+a+mini-replicon+derived+from+the+Corynebacterium+glutamicum+plasmid+pGA1.). 2002; 45:362-367.

2. Schäfer A, Tauch A, Jäger W, Kalinowski J, Thierbach J, Pühler A. Small mobilizable multi-purpose cloning vectors derived from the *Escherichia coli* plasmids pK18 and pK19: selection of defined deletions in the chromosome of *Corynebacterium glutamicum*. Gene.1994;145:69-73.

3. Si MR, Xu YX, Wang TT, Long MX, Ding W, Chen C, et al. Functional characterization of a mycothiol peroxidase in *Corynebacterium glutamicum* that uses both mycoredoxin and thioredoxin system as proton donor for oxidative stress response. Biochem J. 2015;469:45-57.

4. Jakoby M, Ngouoto-Nkili CE, Burkovski A. Construction and application of new *Corynebacterium glutamicum* vectors. Biotechnol Tech.1999;13:437-441.

5. Karimova G, Pidoux J, Ullmann A, Ladant D. A bacterial two-hybrid system based on a reconstituted signal transduction pathway. [Proc Natl Acad Sci U S A](http://www.ncbi.nlm.nih.gov/pubmed/9576956).1998;95:5752-5756.

6. Xu SJ, Peng Z, Cui BY, Wang TT, Song YH, Zhang L, et al. FliS modulates FlgM activity by acting as a non-canonical chaperone to control late flagellar gene expression, motility and biofilm formation in *Yersinia pseudotuberculosis*. [Environ Microbiol](http://www.ncbi.nlm.nih.gov/pubmed/?term=FliS+modulates+FlgM+activity+by+acting+as+a+non-canonical+chaperone+to+control+late+flagellar+gene+expression%2C+motility+and+biofilm+formation).2014;16:1090-1104.
