# Supplementary material for: A thioredoxin-dependent peroxiredoxin Q from Corynebacterium glutamicum plays an important role in defense against oxidative stress
Source: PLoS One. 2018 Feb 13;13(2):e0192674. doi: 10.1371/journal.pone.0192674 (PMC5811025; doi:10.1371/journal.pone.0192674)
Supplement: S2 Table — (DOCX) [file pone.0192674.s002.docx]

**S2 Table. Primers used in this study.**

| **Primers** | **5’-3’ sequence** |  |
| --- | --- | --- |
| PrxQ-F | GGAAGATCTATGACTGAAGTGAAGCGCCTTG( Bgl II) | For cloning *prxQ* and its variants into pXMJ19 and pKT25M-*prxQ* |
| PrxQ-R | ACGCGTCGACTTACGCCGCGGTGATTTCGC(SalI) |  |
| DPrxQ-F1 | CGCGGATCCCGCAAAGGCCATTACACCGCGGCG (BamHI) | To generate pK18*mobsacB* Δ*prxQ* and *prxQ* mutant DNA fragments |
| DPrxQ-R1 | GCGGGTGGGGTATCTCCAACGTCA |  |
| DPrxQ-F2 | TGACGTTGGAGATACCCCACCCGCGCTGGTGCTATTACGCCGCGGTG |  |
| DPrxQ*-*R2 | ACGCGTCGACCTATCCAGTAGCCACGGAGAAGAG (XhoI) |  |
| PPrxQ-F1 | TCCCCCGGGGACCAAGTTGGAAGGCTTGCTG (SmaI) | To generate pK18*mobsacB-P_prxQ_::lacZ* and the 300 bp *prxQ* promoter probe |
| PPrxQ-R1 | ACTAGTCACTTCAGTCATGCACTCCACAAT(SpeI) |  |
| lacZY-F | TATTGTGGAGTGCATGACTGAAGTGACTAGT ATGACCATGATTACGGATTC(SpeI) |  |
| lacZY-R | AAAACTGCAGTTAAGCGACTTCATTCACCTG(PstI) |  |
| PrxQ-C49S-F | GCCAACACCCCAGGT*A*GCACCAAGGAAGCATG | To generate pET28a-*prxQ:C49S* and pXMJ19-*prxQ:C49S* |
| PrxQ-C49S-R | CATGCTTCCTTGGTGCTACCTGGGGTGTTGGC |  |
| PrxQ-C54S-F | GCACCAAGGAAGCA*A*GCGATTTCCGTGATTC | To generate pET28a-*prxQ:C54S* and pXMJ19-*prxQ:C54S* |
| PrxQ-C54S-R | GAATCACGGAAATCGCTTGCTTCCTTGGTGC |  |
| Trx2-F | CGCGGATCCATGGCAACCATCGATGTAACCG(BamHI) | To generate pET28a*-trx2,* pET28a-*trx2:C30S*  and pET28a-*trx2:C33S* |
| Trx2-R | ACGCGTCGACTTATGCCTCTGCAGAACCCTGC (SalI) |  |
| DTrx2-F1 | TCCCCCGGGAAACTCGGAAATCAGAGTGGATGC(Smal) | To generate pET28a-*trx2:C30S* and pET28a-*trx2:C33S* |
| DTrx2-R1 | CCGCTCGAGTGACGATCACCGCGTCCGGGTTCG |  |
| Trx2-C30S-F | CCTGGTGCGGACCT*A*GCCGCCAGTTCGCC | To generate pET28a-*trx2:C30S* |
| Trx2-C30S-R | GGCGAACTGGCGGC*T*AGGTCCGCACCAGG |  |
| Trx2-C33S-F | CCTGGTGCGGACCT*A*GCCGCCAGTTCGCC | To generate pET28a-*trx2:C33S* |
| Trx2-C33S-R | GGCGAACTGGCGGC*T*AGGTCCGCACCAGG |  |
| Trx3-F | CGCGGATCCATGACAAGCAGTGCAAAGTGGTC | To generate pET28a-*trx3* |
| Trx3-R | CCGCTCGAGTTACGGATTCTGCAGCGCCCCTG |  |
| EPrxQ-F | AGAAACCTTAAACCACGAAA | To produce the 300 bp EMSA PrxQ promoter DNA |
| EPrxQ-R | CTCCACAATAGCCGGTGCTC |  |
| Control-F | ATGACCACCAGCAACCCC | To produce the 300 bp EMSA control DNA |
| Control-R | CCCTCGGGGGAACTTCCCGG |  |

Underlined sites indicate restriction enzyme cutting sites added for cloning. Letters in italic denote the mutation sites in overlap PCR for site-directed mutation.
