# Supplementary material for: A thioredoxin-dependent peroxiredoxin Q from Corynebacterium glutamicum plays an important role in defense against oxidative stress
Source: PLoS One. 2018 Feb 13;13(2):e0192674. doi: 10.1371/journal.pone.0192674 (PMC5811025; doi:10.1371/journal.pone.0192674)
Supplement: S3 Table — (DOCX) [file pone.0192674.s003.docx]

**S3 Table. Trx**-**dependent avtivities of PrxQ towards different substrates*^a^*.**

|  | **Trx1** | | | **Trx2** | | |
| --- | --- | --- | --- | --- | --- | --- |
| Substrate | *K*_m_  (μM) | *k*_cat_ (s^-1^) | *k*_cat_*/K*_m_  (10^3^ M^-1^ s^-1^) | *K*_m_  (μM) | *k*_cat_ (s^-1^) | *k*_cat_*/K*_m_  (10^3^ M^-1^ s^-1^) |
| H_2_O_2_ | 220.9±28.1 | 2.11±0.07 | 9.6 | 333.4±27.6 | 1.39±0.03 | 4.2 |
| *t*-BOOH | 316.8 ±47.6 | 0.93±0.05 | 2.9 | 473.9±73.2 | 0.57±0.04 | 1.2 |
| CHP | 93.7±11.1 | 3.77±0.09 | 40.2 | 115.8±22.9 | 2.76±0.04 | 23.8 |

*^a^* Peroxidase assays were performed as described in the Experimental section with the fixed concentration of other components and different peroxide concentrations (0-2000 μM). For determination of Michaelis constant, activities were measured in the reaction mixtures containing 50 mM Tris-HCl buffer (pH 7.5), 2 mM EDTA, 250 μM NADPH, 1 μM PrxQ, 15 μM TrxR, and either 40 μM Trx1 or 40 μM Trx2. The data were analyzed by non-linear regression using the program GraphPad Prism 5 and presented as means of the values obtained from three independent assays.
