# Supplementary figures and images for: A thioredoxin-dependent peroxiredoxin Q from Corynebacterium glutamicum plays an important role in defense against oxidative stress
Source: PLoS One. 2018 Feb 13;13(2):e0192674. doi: 10.1371/journal.pone.0192674 (PMC5811025; doi:10.1371/journal.pone.0192674)

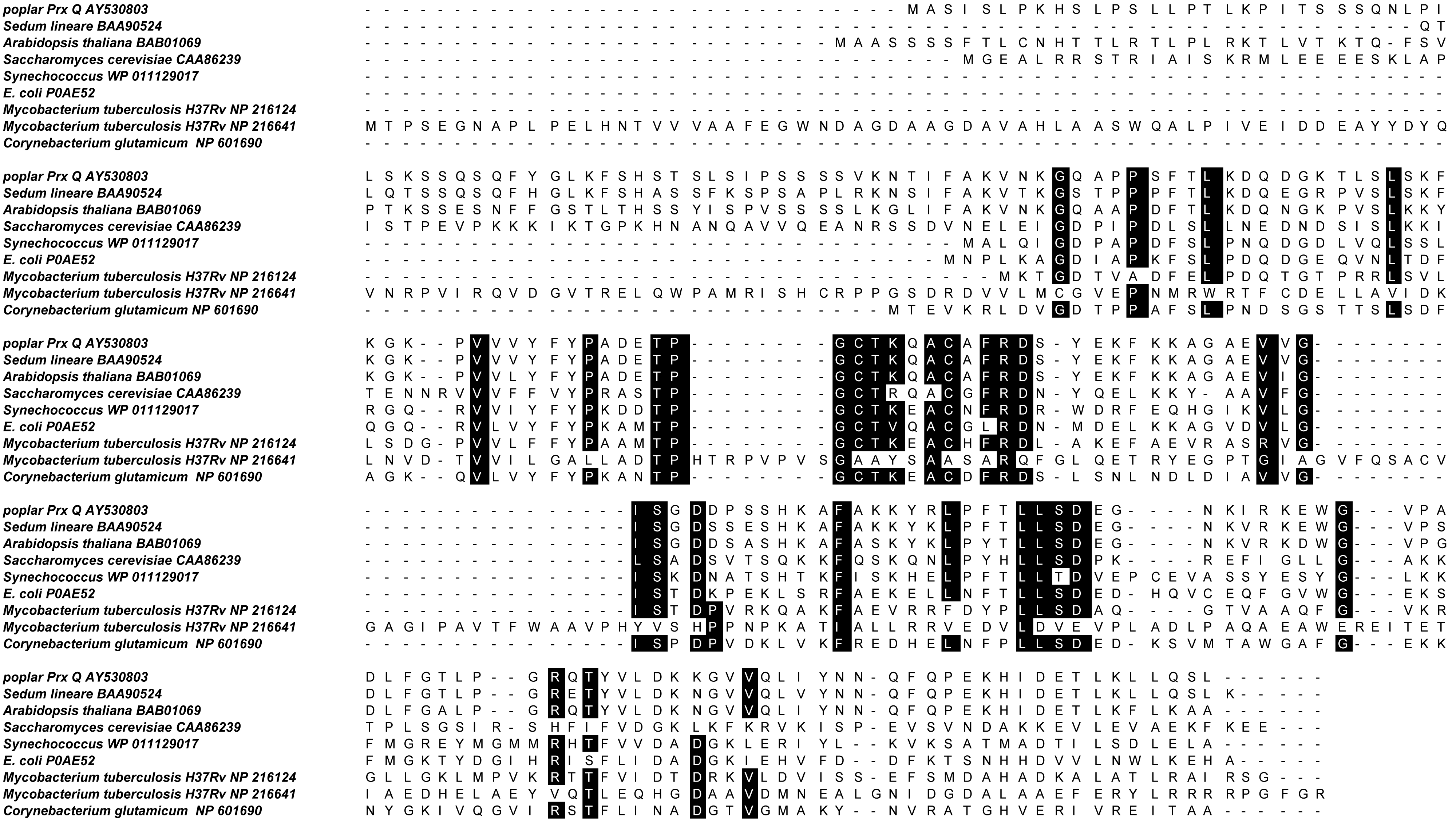

Supplement: S1 Fig — The lack background showed the strong conserved Cys in positions 49 and 53 (C. glutamicum PrxQ numbering). Accession numbers: C. glutamicum ATCC 13032 (NP_601690); E. coli (P0AE52); poplar (AY530803); Sedum lineare (BAA90524); Arabidopsis thaliana (BAB01069); accharomyces cerevisiae (CAA86239); Nostoc sp. PCC 7120 (BAB74202); Agrobacterium fabrum str. C58 (NP_354814); Rhizobium etli (WP_011425522); Synechococcus (WP_011129017); Mycobacterium tuberculosis (NP_216124); Mycobacterium tuberculosis (NP_216641). (TIF) [file pone.0192674.s004.tif]

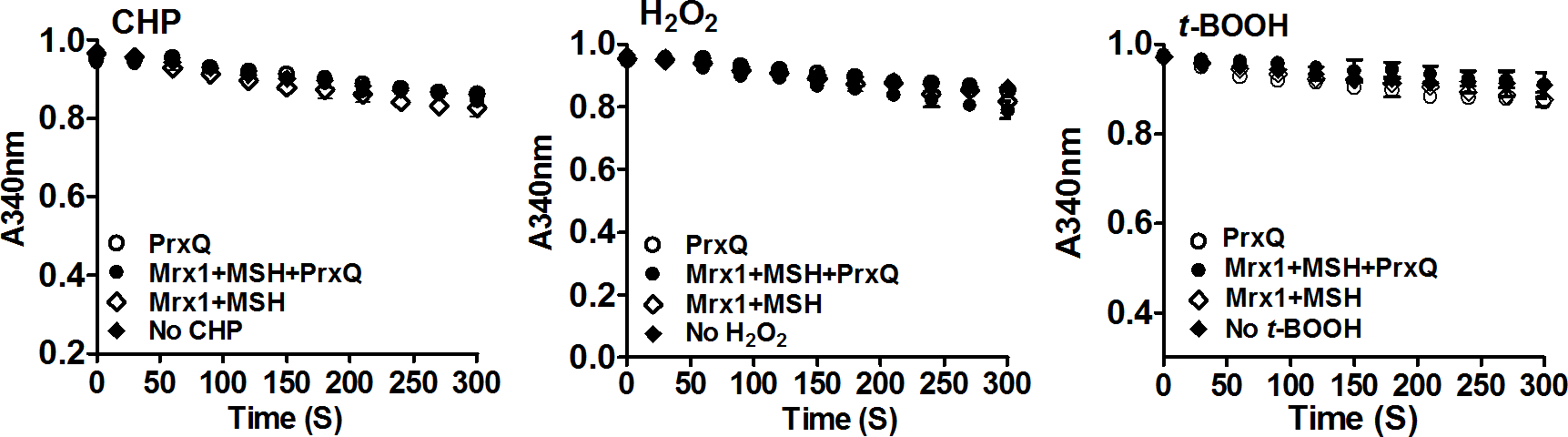

Supplement: S2 Fig — The reduction of peroxides was recorded by measuring the decrease of NADPH oxidation at 340 nm. The reactions omitted the Mrx1 electron pathway, PrxQ, or peroxide served as negative controls. Similar results were obtained in three independent experiments, and data shown are from one representative experiment done in triplicate. (TIF) [file pone.0192674.s005.tif]

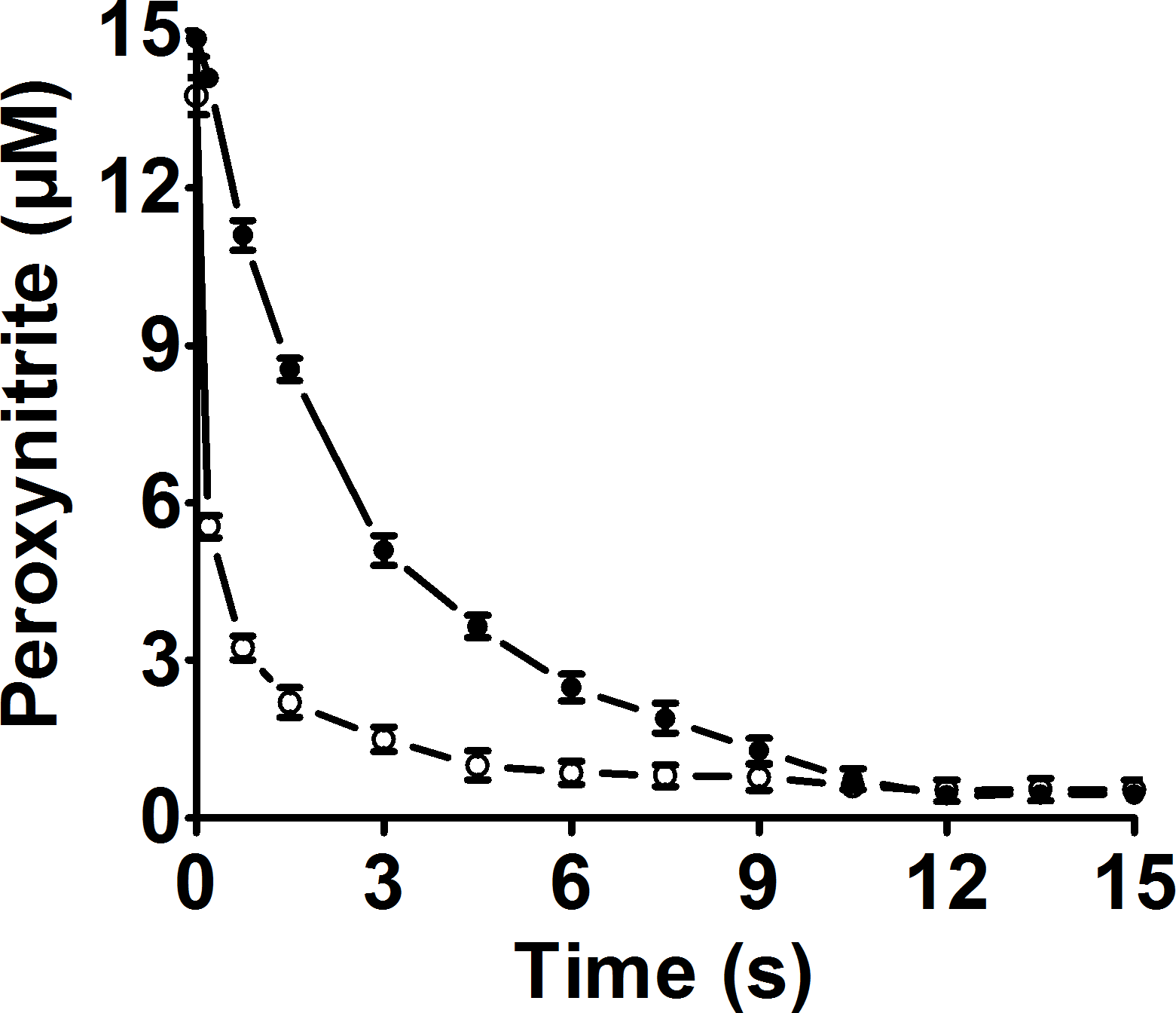

Supplement: S3 Fig — Time trace of peroxynitrite decay in absence and presence of PrxQ B (15 μM) at pH 7.4 at 25°C. The data were presented as means of the values obtained from three independent assays. (TIF) [file pone.0192674.s006.tif]

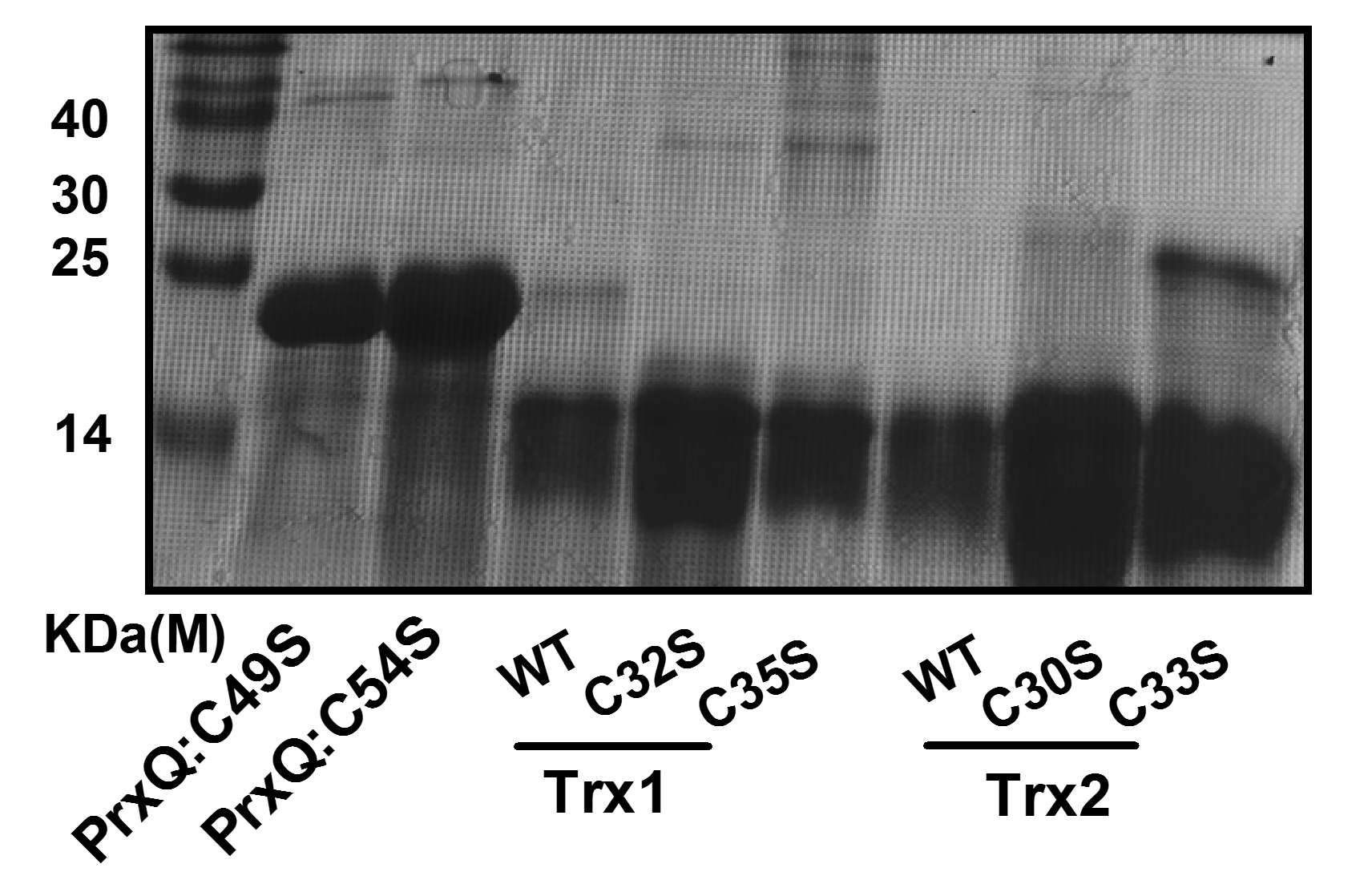

Supplement: S4 Fig — Non-reducing 15% SDS-PAGE showing states of PrxQ (Its variants), Trx1 (WT and its variants) and Trx2 (WT and its variants) (20 μM) in the presence of 50 μM H2O2. (TIF) [file pone.0192674.s007.tif]

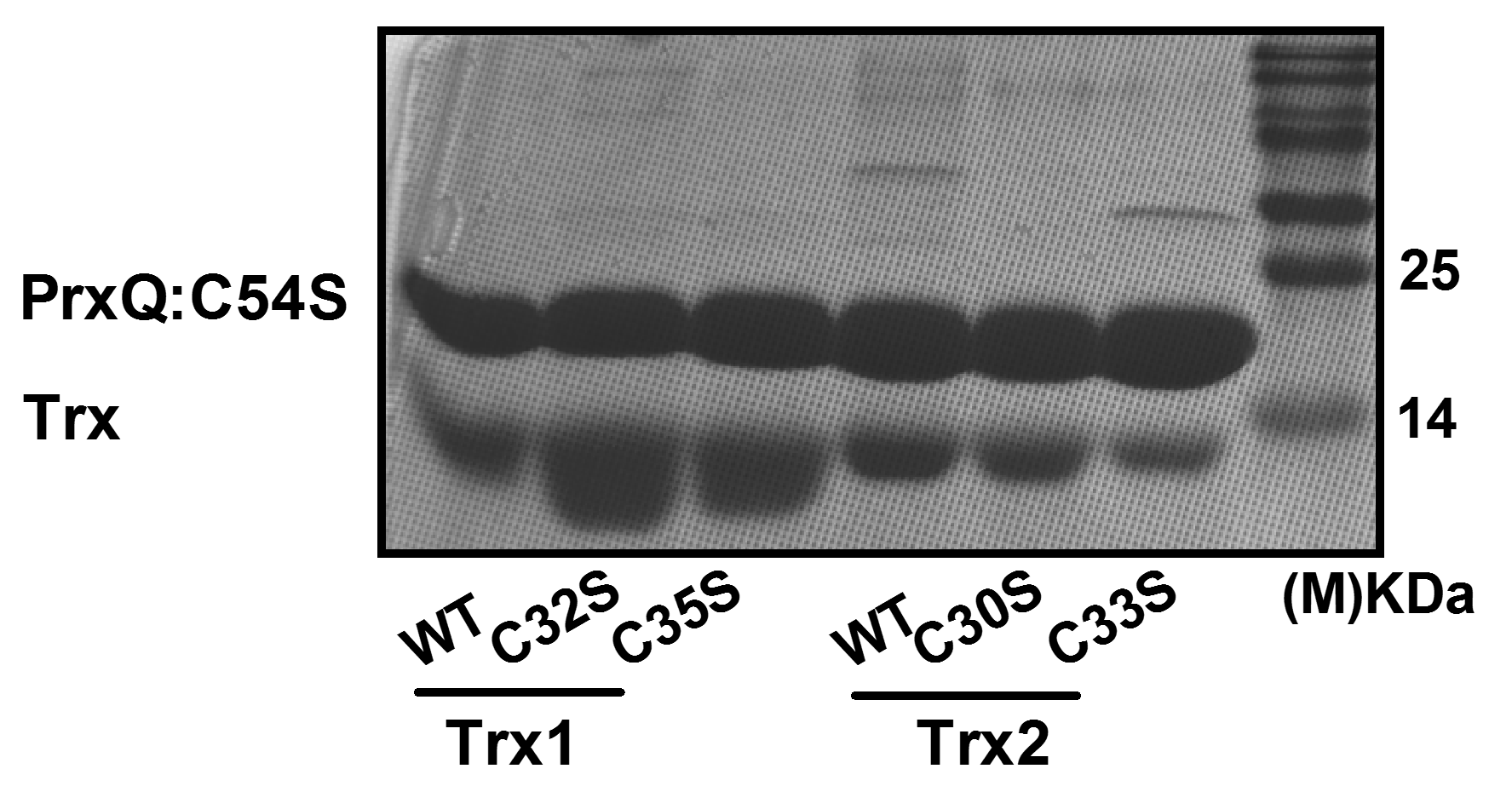

Supplement: S5 Fig — M: protein molecular weight marker. (TIF) [file pone.0192674.s008.tif]

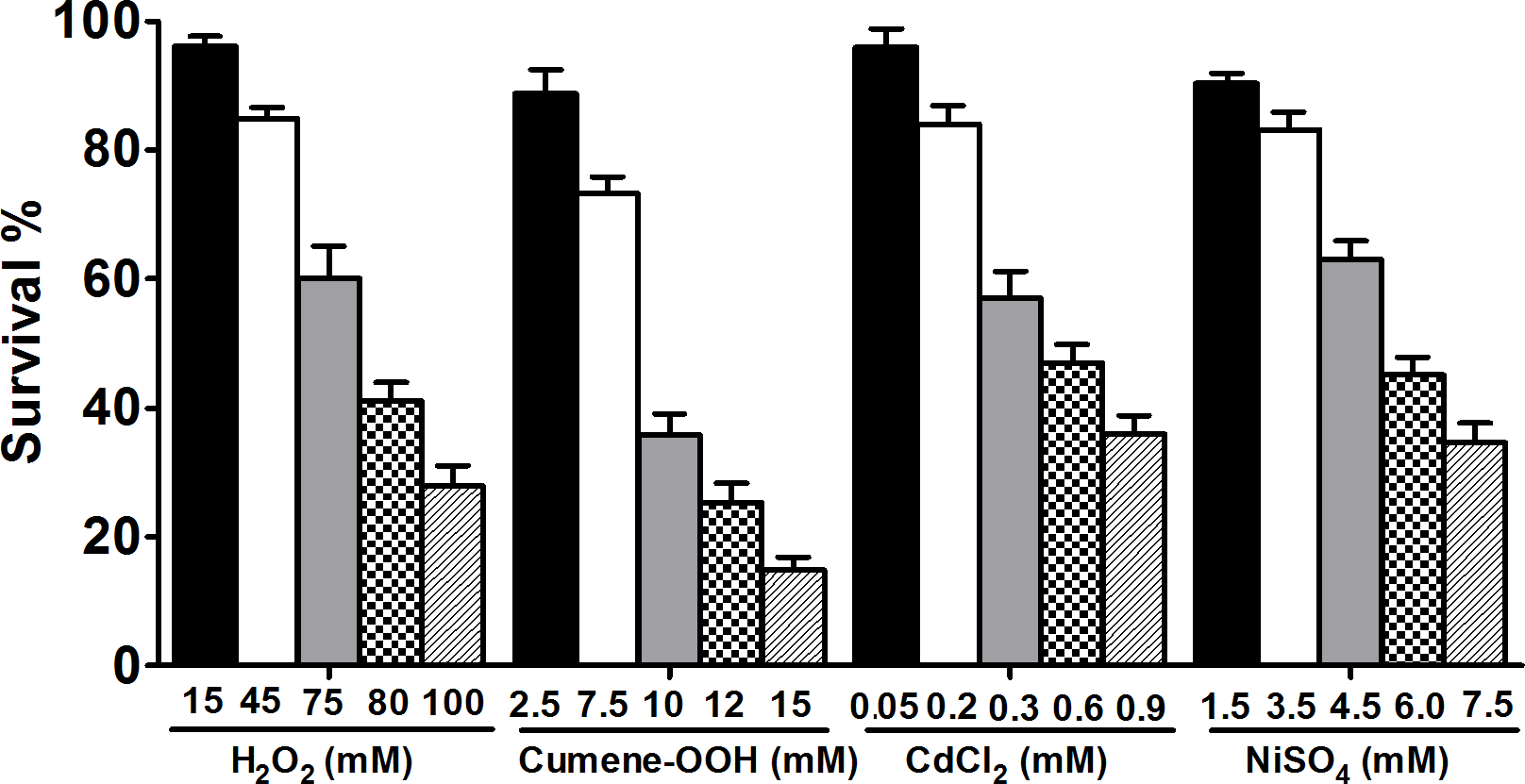

Supplement: S6 Fig — C. glutamicum wild type was grown in LB medium to an OD600 of 1.0 and exposed to different concentrations of various peroxides at 30°C for 30 min. After treatment, the cultures were serially diluted, spreaded on LB plates and incubated at 30°C for 36 h. Survival percentages were calculated as [(CFU ml-1 with stress)/(CFU ml-1 without stress)]×100. Mean values with standard deviations (error bars) from at least three repeats are shown. (TIF) [file pone.0192674.s009.tif]
